# Two-Photon Bidirectional Control and Imaging of Neuronal Excitability with High Spatial Resolution *In Vivo*

**DOI:** 10.1016/j.celrep.2018.02.063

**Published:** 2018-03-13

**Authors:** Angelo Forli, Dania Vecchia, Noemi Binini, Francesca Succol, Serena Bovetti, Claudio Moretti, Francesco Nespoli, Mathias Mahn, Christopher A. Baker, McLean M. Bolton, Ofer Yizhar, Tommaso Fellin

**Affiliations:** 1 Optical Approaches to Brain Function Laboratory, Istituto Italiano di Tecnologia, Genova 16163, Italy; 2 Department of Neurobiology, Weizmann Institute of Science, Rehovot 76100, Israel; 3 Disorders of Neural Circuit Function, Max Planck Florida Institute for Neuroscience, Jupiter 33458, FL, USA

**Keywords:** optogenetics, two-photon excitation, digital holography, patterned illumination, two-photon imaging

## Abstract

Sensory information is encoded within the brain in distributed spatiotemporal patterns of neuronal activity. Understanding how these patterns influence behavior requires a method to measure and to bidirectionally perturb with high spatial resolution the activity of the multiple neuronal cell types engaged in sensory processing. Here, we combined two-photon holography to stimulate neurons expressing blue light-sensitive opsins (ChR2 and GtACR2) with two-photon imaging of the red-shifted indicator jRCaMP1a in the mouse neocortex *in vivo*. We demonstrate efficient control of neural excitability across cell types and layers with holographic stimulation and improved spatial resolution by opsin somatic targeting. Moreover, we performed simultaneous two-photon imaging of jRCaMP1a and bidirectional two-photon manipulation of cellular activity with negligible effect of the imaging beam on opsin excitation. This all-optical approach represents a powerful tool to causally dissect how activity patterns in specified ensembles of neurons determine brain function and animal behavior.

## Highlights

• High-resolution bidirectional control of cell activity in the intact mouse brain
• High-resolution manipulation is effective across cortical cell types and layers
• Simultaneous all-optical imaging and bidirectional manipulation
• Minimal crosstalk between imaging and opsin activation

## Graphical Abstract

**Figure:**
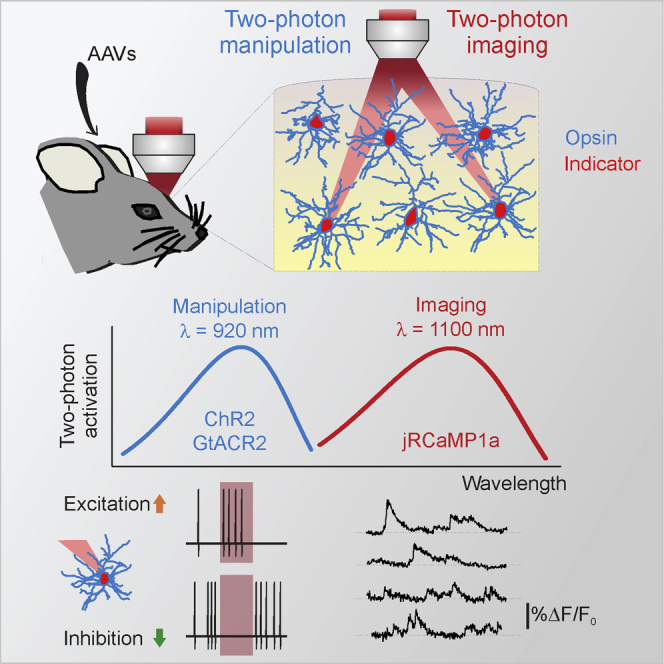

## Teaser

Forli et al. developed an all-optical method to image and bidirectionally manipulate brain networks with high spatial resolution and minimal crosstalk in the intact mammalian brain. They validate the method across cell types and layers in the mouse neocortex.

## Introduction

Within brain circuits, information about sensory stimuli is encoded in complex spatial and temporal patterns of activity distributed across cells (Kampa et al., 2011, Ohki et al., 2005, Sawinski et al., 2009). For example, population recordings, combined with statistical analysis, showed that specific features of sensory stimuli elicit temporally structured responses in specific ensembles of neurons (Carrillo-Reid et al., 2016, Miller et al., 2014). However, using statistical analysis and correlative evidence to causally test which sensory features are encoded in neural circuits and how this information is used to drive behavior may prove difficult (Panzeri et al., 2017). To achieve this goal, we would ideally need a method to monitor and bidirectionally perturb the activity of multiple neurons maintaining single-cell resolution. With such a technique, it would be possible to study how the concerted activity of identified neurons contributes to network function by activating or inactivating populations of functionally characterized neurons with cellular resolution.

Optical approaches, in particular two-photon microscopy, hold promise to achieve this goal. Moreover, wave-front engineering methods (Emiliani et al., 2005) using digital holography largely extended the potential of two-photon microscopy for imaging (Bovetti et al., 2017, Dal Maschio et al., 2010, Ducros et al., 2013, Moretti et al., 2016, Nikolenko et al., 2008, Quirin et al., 2013, Yang et al., 2015, Yang et al., 2016) and photostimulation applications (Chaigneau et al., 2016, Dal Maschio et al., 2017, Lutz et al., 2008, Packer et al., 2012, Papagiakoumou et al., 2010, Papagiakoumou et al., 2013, Szabo et al., 2014). In parallel to these improvements in optics, the last decade witnessed the development of a large toolbox of light-sensitive molecules to monitor and manipulate the activity of neurons, including opsins such as channelrhodopsin-2 (ChR2) (Boyden et al., 2005, Nagel et al., 2003), C1V1 (Prakash et al., 2012, Yizhar et al., 2011), and Guillardia theta Anion Channelrhodopsins (GtACRs) (Govorunova et al., 2015) and functional indicators such as GCaMPs (Chen et al., 2013) and RCaMPs (Dana et al., 2016). Combining advanced two-photon approaches with the use of these bioengineered molecules, it became possible to perform simultaneous functional imaging of GCaMP signals and stimulation of various opsins (e.g., C1V1) with high spatial resolution in the rodent brain *in vivo* (Carrillo-Reid et al., 2016, Packer et al., 2015, Rickgauer et al., 2014, Yang et al., 2018) and in other experimental systems (Dal Maschio et al., 2017, Förster et al., 2017, Hernandez et al., 2016). However, several limitations need to be overcome to efficiently apply these approaches. First, crosstalk between imaging and photostimulation needs to be minimized. For instance, the red-shifted channelrhodopsin C1V1 is maximally activated using 540 nm light (Yizhar et al., 2011), but it is still more than half-maximally activated by 470 nm light. This shoulder toward shorter wavelengths (Yizhar et al., 2011) typical for red-shifted opsins (Venkatachalam and Cohen, 2014) is reflected in their two-photon absorption spectra (Chaigneau et al., 2016, Prakash et al., 2012, Ronzitti et al., 2017) and may lead to non-negligible neuronal depolarization during two-photon GCaMP imaging (Packer et al., 2015, Rickgauer et al., 2014, Ronzitti et al., 2017, Yang et al., 2018). This undesired effect worsens when opsins with slow off kinetics and high-amplitude photocurrents, which are the preferred choice for two-photon activation of neurons with the raster or spiral scanning approach, are used (Chaigneau et al., 2016, Dal Maschio et al., 2017). Second, while published data demonstrated cellular resolution two-photon activation of neurons (Packer et al., 2015, Rickgauer et al., 2014), evidence for efficient patterned two-photon inhibition, as well as all-optical imaging and high-resolution inhibitory manipulation *in vivo*, is still to be provided. Third, whether single-cell two-photon optogenetics can be efficiently applied across the various cell types that are engaged during sensory stimulation and that differ in morphology, biophysical properties, and cortical depth is unclear.

Here we developed an experimental approach to address all of these challenges in the mouse cortex *in vivo*. We combined digital holography to stimulate blue light-sensitive opsins with two-photon imaging of a red-shifted functional indicator. We show that holographic illumination of ChR2 (Nagel et al., 2005) with extended shapes can be used to efficiently stimulate various cellular populations, including principal cells and different interneuron types, in cortical layer 2/3 and in layer 4, the main thalamorecipient lamina in sensory cortex (Feldmeyer et al., 2013). We then characterized the two-photon excitability of the chloride-permeable channelrhodopsin GtACR2 in slice preparation and showed that it efficiently decreases neuronal firing with high spatial resolution *in vivo* upon holographic illumination. Finally, combining soma-targeting of opsins (ChR2 and GtACR2), which improved the spatial resolution of stimulation, with the use of the red-shifted calcium indicator jRCaMP1a, we provide a proof-of-principle demonstration of simultaneous two-photon imaging and bidirectional holographic stimulation of cells with negligible effect of the imaging beam on opsin excitation.

## Results

To test whether using a blue light-sensitive channelrhodopsin would lead to a reduction in the undesired cross-activation during activity reporter imaging, we expressed ChR2 and C1V1_(T/T)_ in cultured hippocampal neurons and characterized the photocurrent evoked by two-photon scanning at the wavelengths typically used for calcium imaging of green and red calcium indicators (920 and 1,080 nm, respectively) (Figure S1). ChR2-expressing neurons showed lower relative peak current amplitudes when scanned at 1,080 nm than did C1V1-expressing neurons scanned at 920 nm (Figures S1C–S1E). The relative average photocurrent evoked during scanning at 1,080 nm was also higher for C1V1 (Figures S1C and S1F). Increasing the raster-scanning rate led to further elevation of C1V1 activation (Figures S1C and S1F) because of the slower closing kinetics of C1V1 (Yizhar et al., 2011). Conversely, increasing the raster scanning rate when recording from ChR2-expressing cells did not increase photocurrents (Figures S1D and S1F), consistent with its faster off kinetics.

### High Spatial Resolution Two-Photon Holographic Stimulation *In Vivo*

**Figure 1:**
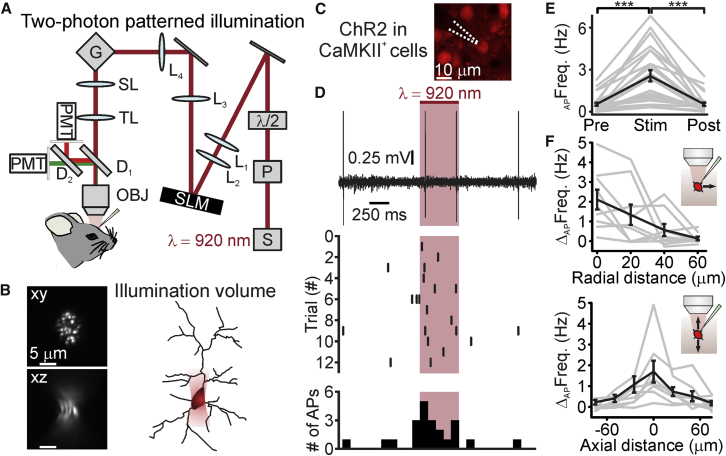
Two-Photon Holographic Stimulation of ChR2-Expressing Cells with Extended Shapes *In Vivo* (A) Optical setup for holographic illumination. S, laser source; P, Pockels cell; λ/2, half-wave plate; L_1–4_, lenses; SLM, spatial light modulator; G, galvanometric mirrors; SL, scan lens; TL, tube lens; D_1–2_, dichroic mirrors; PMT, photomultiplier tube; OBJ, objective. (B) Left: intensity profiles in the focal plane (xy, top) and along the axial direction (xz, bottom) of an extended shape illuminating a thin (thickness: ∼150 nm) fluorescent layer and generated with the setup displayed in (A). λ_exc_ = 920 nm. Right: schematic of the single-cell holographic stimulation paradigm. An elliptical shape was drawn on the soma of opsin-expressing neurons, resulting in an extended illumination volume covering the cell body of the target cell. (C) Two-photon image of a layer 2/3 cortical neuron co-expressing ChR2-mCherry and tdTomato. The cell was targeted for simultaneous holographic stimulation and juxtasomal electrophysiological recording, monitoring the fluorescence of tdTomato *in vivo*. The dotted lines indicate the recording pipette. (D) Top: electrophysiological trace recorded before, during, and after holographic stimulation (red bar, laser power: 80 mW). Middle: raster plot showing cell response over consecutive trials for the same neuron displayed in the top panel. Bottom: AP distribution for the trials shown in the middle panel (time bin: 100 ms). (E) Firing frequency before (Pre), during (Stim), and after (Post) holographic stimulation of ChR2-expressing layer 2/3 neurons. p = 2E−9, Friedmann test with Dunn’s correction, N = 28 cells from 10 mice. Average laser power: 54 mW, range: 30–92 mW. (F) Firing frequency increase versus displacement of the excitation volume in the radial (top) and axial (bottom) directions during holographic illumination of layer 2/3 cells expressing ChR2-eYFP. Top: N = 9 cells from 6 mice. Bottom: N = 8 cells from 6 mice. Black line represents the average and SEM, and individual experiments are depicted in gray. ^∗^p < 0.05; ^∗∗^p < 0.01; ^∗∗∗^p < 0.001. See also Figures S1–S3 and Tables S1–S3.

To stimulate neurons with high spatial resolution *in vivo*, we used a liquid crystal spatial light modulator (SLM)-based holographic module, which was integrated in a commercial laser scanning two-photon microscope (Figure 1A) (Dal Maschio et al., 2010, Dal Maschio et al., 2011), and we programmed the holographic module (see Experimental Procedures) to project on the sample plane elliptical shapes that were centered on the cell body of target neurons (Figure 1B; Figure S2). To validate our approach, we performed simultaneous two-photon targeted juxtasomal recordings and photostimulation experiments in anesthetized mice in layer 2/3 principal neurons co-expressing ChR2 and the red fluorescent protein tdTomato, which facilitated targeting neurons under the microscope (Figure 1C). Once a stable electrophysiological recording was achieved from an opsin-positive neuron (see Experimental Procedures for definition), a high-resolution image was acquired and an elliptical shape (ellipse axis: 7–16 μm) was projected on the cell body of the recorded neuron. Significant increase in action potential (AP) firing frequency was observed upon two-photon holographic illumination with extended elliptical shapes (stimulus duration: 500 ms; stimulus power: 30–92 mW/cell; λ_exc_ = 920 nm) (Figures 1D and 1E). To verify that the observed effect depended on opsin activation, not on membrane depolarization due to direct two-photon stimulation (Hirase et al., 2002), we performed similar experiments in opsin-negative cells (Figure S3). We found that holographic illumination with extended shapes of the same spatial profile and light intensity did not modify the membrane potential or the AP firing rate of recorded opsin-negative neurons *in vivo* (Figures S3C and S3D).

**Figure 2:**
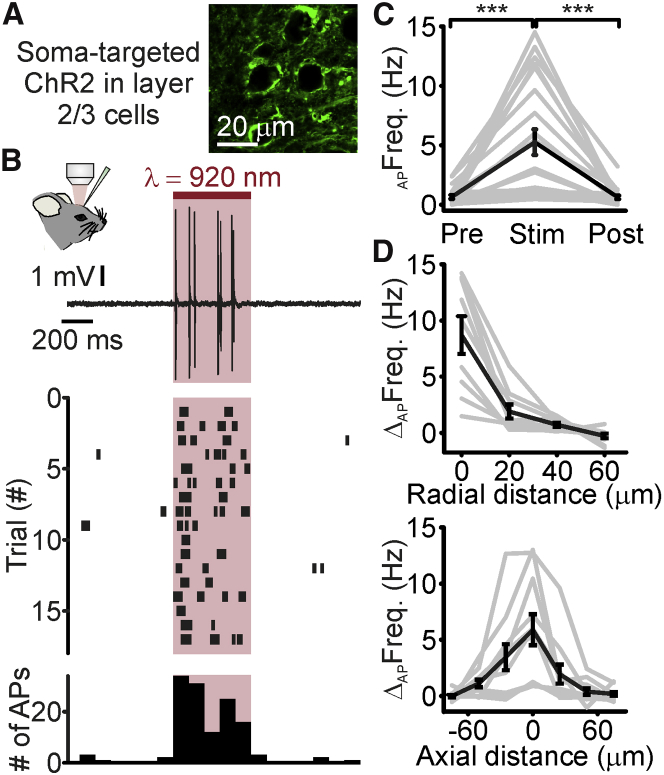
Opsin Somatic Targeting Increases the Spatial Resolution of Holographic Stimulation *In Vivo* (A) Confocal image of layer 2/3 cells expressing soma-targeted ChR2-eYFP (green). (B) Top: juxtasomal electrophysiological trace recorded Pre, during (Stim), and Post holographic stimulation (red bar, laser power: 30 mW; λ_exc_ = 920 nm) of a cortical layer 2/3 neuron expressing the soma-targeted ChR2 *in vivo*. Middle: raster plot showing cell response over consecutive trials for the same cell displayed in the top panel. Bottom: AP distribution for the trials shown in the middle panel (time bin: 100 ms). (C) Average firing frequency Pre, during (Stim), and Post holographic stimulation of layer 2/3 cells expressing the soma-targeted ChR2. p = 1.3E−7, Friedmann test with Dunn’s correction, N = 21 cells from 6 mice. Laser power: 30 mW. (D) Firing frequency increase versus displacement in the radial (top) and axial (bottom) directions during holographic illumination. Top: N = 9 cells from 3 mice. Bottom: N = 11 cells from 3 mice. In this figure, the black line represents the average and SEM, individual experiments are depicted in gray. See also Figure S4 and Tables S1–S3.

To evaluate the spatial resolution of our stimulation method, we measured the spiking response to holographic stimulation of opsin-positive neurons while incrementally shifting the stimulation shape in the radial and axial direction (Figure 1F). We found spatial constants (see Experimental Procedures for definition) of ∼20, ∼32, and ∼16 μm in the radial, axial_up_, and axial_down_ directions, respectively. Targeting ChR2 to the soma (Figure 2) increased the average spiking response in the illuminated neuron (ChR2: Δ_AP_Freq = 1.2 ± 0.3 Hz, N = 15 cells from 6 mice; soma-targeted ChR2: Δ_AP_Freq = 4.6 ± 1.0 Hz, p = 2.2E−2, Mann-Whitney test, N = 21 neurons from 6 mice; stimulus power: 30 mW for both ChR2 and soma-targeted ChR2). Somatic targeting of ChR2 improved the spatial resolution of holographic stimulation compared to non-soma-targeted opsins, decreasing the axial_up_ (13 and 32 μm for soma-targeted and non-soma-targeted opsins, respectively; p = 1.4E−2, unpaired Student’s t test) spatial constant (Table S1). Confocal analysis of fixed sections from injected animals confirmed restricted expression in the somatic and perisomatic compartments with the soma-targeted ChR2 compared to the non-soma-targeted ChR2 (Figure S4). Table S1 also shows the density of opsin-expressing cells under our experimental conditions. The values of the radial and axial space constants of photostimulation resolution normalized to the soma diameter of the stimulated cells are shown in Table S2.

### Two-Photon Holographic Stimulation across Cortical Cell Types and Layers

**Figure 3:**
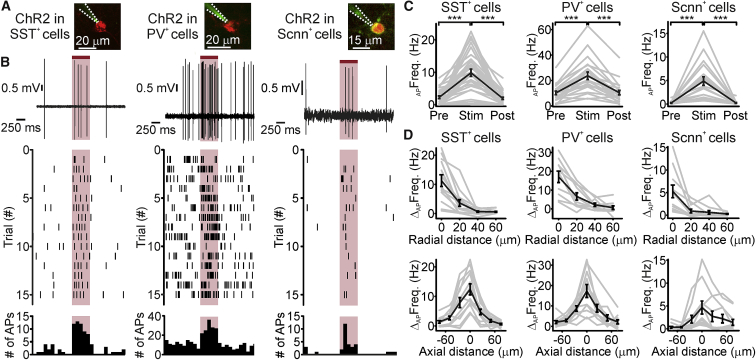
Two-Photon Holographic Stimulation across Cell Types and Layers *In Vivo* (A) Two-photon image of one layer 2/3 SST^+^ interneuron (left) and one layer 2/3 PV^+^ interneuron (middle) expressing ChR2-mCherry (red). One layer 4 Scnn^+^ neuron expressing ChR2-eYFP (green), together with tdTomato (red), is shown on the right. Neurons were recorded in the juxtasomal configuration with a glass pipette (dotted white line) filled with Alexa Fluor 488 (green) *in vivo*. (B) Top: electrophysiological traces recorded Pre, during (Stim), and Post holographic stimulation (red bar) for one SST^+^ cell (left), one PV^+^ cell (middle), and one Scnn^+^ cell (right). λ_exc_ = 920 nm. Laser power: 30 mW for SST^+^ and PV^+^ cells and 50 mW for Scnn^+^ neurons. Middle: raster plot showing cell response over consecutive trials for the same neurons displayed in the top panel. Bottom: AP distribution for the trials shown in the middle panel (time bin: 100 ms) for all cell types (SST^+^, left; PV^+^, middle; Scnn^+^, right). (C) Average firing frequency Pre, during (Stim), and Post holographic stimulation of ChR2-expressing layer 2/3 SST^+^ neurons (left), layer 2/3 PV^+^ neurons (middle), and layer 4 Scnn^+^ neurons (right). SST^+^ cells: p = 1.3E−9, Friedmann test with Dunn’s correction, N = 31 cells from 7 mice. PV^+^ cells: p = 1.2E−7, ANOVA test with Bonferroni’s correction, N = 22 cells from 7 mice. Scnn^+^ cells p = 5.2E−7, Friedmann test with Dunn’s correction, N = 19 cells from 8 mice. Laser power: 30 mW for SST^+^ and PV^+^ cells and 50 mW for Scnn^+^ cells. (D) Firing frequency increase versus displacement in the radial (top) and axial (bottom) directions during holographic illumination for layer 2/3 SST^+^ neurons (left), layer 2/3 PV^+^ neurons (middle), and layer 4 Scnn^+^ neurons (right). SST^+^ cells: top, N = 13 cells from 3 mice; bottom, N = 12 cells from 3 mice. PV^+^ cells: top, N = 10 cells from 4 mice; bottom, N = 11 cells from 4 mice. Scnn^+^ cells: top and bottom, N = 11 cells from 5 mice. In this figure, the black line represents the average and SEM, individual experiments are depicted in gray. See also Figure S5 and Tables S1–S3.

We investigated whether holographic stimulation could be efficiently applied to cell types other than layer 2/3 excitatory neurons. To this end, we first expressed ChR2 in two major subpopulations of cortical interneurons in layer 2/3, the somatostatin-positive (SST^+^) and the parvalbumin-positive (PV^+^) cells. Using simultaneous photostimulation and two-photon targeted juxtasomal recordings *in vivo*, we found that illumination with an extended shape (stimulus power: 30 mW/cell) increased the firing rate of targeted interneurons (Figures 3A–3C, left and middle). We then expressed ChR2 selectively in sodium channel, non-voltage-gated 1 alpha-positive (Scnn^+^) excitatory neurons of layer 4, the main thalamorecipient cortical population of the sensory cortex. We found that holographic illumination (stimulus power: 50 mW/cell) increased the spike rate of layer 4 Scnn^+^ neurons (Figures 3A–3C, right). In all excitatory neurons recorded in layer 2/3 (Figures 1 and 2) and layer 4 (Figure 3), the spontaneous firing rates before and after photostimulation were not significantly different (Table S3). In all cell types the response to photostimulation depended upon the illumination power (Figure S5).

### High Spatial Resolution Two-Photon Holographic Inhibition *In Vivo*

**Figure 4:**
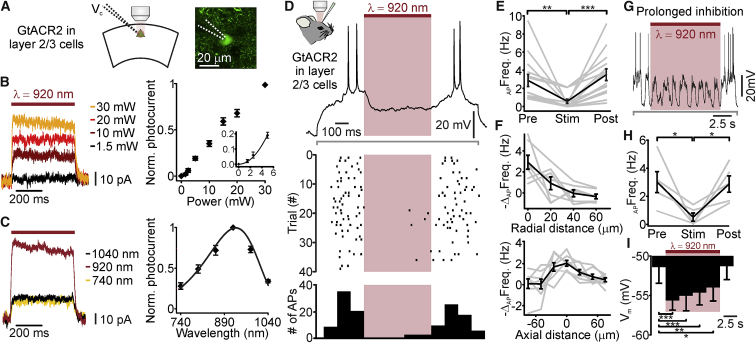
Two-Photon Holographic Activation of GtACR2 Allows Optogenetic Inhibition with High Spatial Resolution *In Vitro* and *In Vivo* (A) Left: schematic of the experimental configuration for slice recording. Neurons expressing GtACR2 were recorded in voltage-clamp configuration (V_c_) and held at −50 mV while holographic illumination with an elliptical shape was performed. Chloride equilibrium potential: −68 mV. Right: two-photon image of a layer 2/3 neuron expressing GtACR2-eGFP that was recorded in voltage-clamp configuration. The glass pipette (dotted lines) was filled with Alexa Fluor 488 (green cytosolic signal). (B) Left: GtACR2-mediated photocurrents evoked by holographic illumination at different illumination powers. Traces are averages of 3 stimulation trials. Right: average peak photocurrent evoked by holographic illumination at various laser powers. λ_exc_ = 920 nm. In each cell, photocurrent values were normalized to the maximal photocurrent recorded at 30 mW light power. The inset displays the nonlinear dependence of photocurrents on the laser power at low power values. N = 7–11 cells from 2 to 3 mice. (C) Left: traces showing GtACR2 photocurrents evoked by holographic illumination at different wavelengths. Traces are averages of 4 stimulation trials. Average laser power: 25 mW. Right: average GtACR2 peak photocurrent evoked by holographic illumination as a function of the stimulation wavelength. Photocurrent values were normalized to the peak photocurrent at 920 nm. Values were fitted with a three-parameter Weibull function. N = 9 cells from 4 mice. Laser power: 20–30 mW. (D) Top: membrane potential of one layer 2/3 GtACR2-expressing neuron recorded in whole-cell configuration *in vivo* during simultaneous current injection (gray line below the trace; current amplitude: 70 pA) and two-photon holographic illumination (red bar). Laser power: 80 mW; stimulus duration: 500 ms. Inset: schematic of the experimental configuration *in vivo*. Middle: raster plot showing cell response over consecutive trials for the same cell displayed in the top panel. Bottom: AP distribution for the trials shown in the middle panel (time bin: 100 ms). (E) Average firing frequency Pre, during (Stim), and Post holographic stimulation of GtACR2-expressing layer 2/3 neurons. Holographic illumination was performed while injecting a small depolarizing current. Average current amplitude: 74 pA, range: 50–100 pA. p = 1.8E−5, Friedmann test with Dunn’s correction, N = 14 cells from 7 mice. Average laser power: 50 mW, range: 10–80 mW. (F) Firing frequency decrease versus displacement of the excitation volume in the radial (top) and axial (bottom) directions during holographic illumination. Top: N = 7 cells from 4 mice. Bottom: N = 8 cells from 5 mice. (G) Same as (D), but for a prolonged period of illumination (10 s). Injected current amplitude: 70 pA; laser power: 80 mW. (H) Same as (E) but for prolonged illumination. Average injected current amplitude: 50 pA, range: 30–70 pA. p = 9.4E−3, ANOVA with Bonferroni’s correction, N = 5, from 2 mice. Laser power: 80 mW. (I) Membrane potential Pre, during (Stim), and Post prolonged inhibition of layer 2/3 neurons with holographic stimulation of GtACR2 (time bins: 2.5 s). p = 7E−6, ANOVA test with Bonferroni’s correction, N = 5, from 2 mice. Laser power: 80 mW. In this figure, the black line represents the average and SEM, individual experiments are depicted in gray. See also Tables S1 and S2.

Our previous data demonstrate that holographic stimulation with extended shapes can be used for activation of neurons with high spatial resolution *in vivo*. We determined whether holographic illumination could also be used for efficient two-photon optogenetic inhibition with similar spatial precision. To address this question, we focused on GtACR2, a chloride-permeable channelrhodopsin (Govorunova et al., 2015). Although the two-photon excitability of GtACR2 has not yet been reported, we reasoned that its large photocurrent, high light sensitivity, and blue light-sensitive, single-photon absorption spectrum made it a good candidate for two-photon holographic stimulation at λ < 1,000 nm. In addition, expression of GtACR2 has been reported to be well tolerated by neurons (Govorunova et al., 2015). We first expressed this inhibitory opsin in the cortex and recorded GtACR2-mediated photocurrents in a patch-clamp, voltage-clamp configuration from opsin-positive cells (see Experimental Procedures for definition) in acute brain slices (Figure 4A). We found that holographic illumination of GtACR2-expressing neurons with an elliptical shape targeted to the cell body of the recorded cell (λ_exc_ = 920 nm; stimulus power: 30 mW; stimulus duration: 500 ms) triggered clear outward currents (range: 6–112 pA; holding potential: −50 mV; chloride equilibrium potential: −68 mV). Peak amplitude of photocurrents increased with power (Figure 4B) and showed a nearly power-squared dependence for low power values (Figure 4B, inset). Moreover, while keeping light power density constant, we performed holographic two-photon illumination at different light wavelengths (range: 740–1,040 nm). We found that GtACR2 photocurrents had large peak amplitude at 920 nm and decreased for longer and shorter wavelengths (Figure 4C). We thus concluded that GtACR2 can be efficiently stimulated through a two-photon absorption process, that holographic illumination triggers clear inhibitory photocurrents in opsin-expressing neurons, and that the two-photon absorption spectrum of GtACR2 shows a clear peak ∼920 nm.

We then asked whether holographic stimulation of GtACR2 could be used to decrease neural excitability with high spatial resolution *in vivo*. To this end, we performed whole-cell, current-clamp recordings from layer 2/3 cortical neurons expressing GtACR2 in anesthetized mice (Figures 4D–4I). We found that illumination with an extended shape (stimulus power: 10–80 mW/cell; stimulus duration: 500 ms) while a small depolarizing current was injected (current amplitude: 74 pA) led to a significant hyperpolarization of the cell (average membrane potential before [Pre]: −46.2 ± 1.1 mV, during [Stim]: −50.1 ± 1.1 mV, after [Post]: −44.4 ± 1.3 mV; p = 2E−15, ANOVA test with Bonferroni’s correction, N = 14 from 7 mice). Moreover, we found that holographic illumination decreased cellular firing induced by a small current injection (Figures 4D and 4E). We measured the spiking response of GtACR2-positive neurons to holographic illumination while incrementally shifting the stimulation shape in the radial and axial directions (Figure 4F). We found spatial constants of ∼11, ∼33, and ∼29 μm in the radial, axial_up_, and axial_down_ directions, respectively (Table S1). Prolonged illumination (stimulus duration: 10 s) of GtACR2-expressing neurons decreased the firing rate (Figures 4G and 4H), and it hyperpolarized the membrane potential of the illuminated cell for the duration of the light stimulus (Figure 4I). The values of the cell resting membrane potential Pre- and Post-photostimulation were not significantly different (average resting membrane potential Pre: −61.2 ± 2.2 mV, Post: −62.2 ± 1.5 mV; p = 0.43, Student’s t test, N = 5 from 2 mice).

### Simultaneous Two-Photon Imaging of Red-Shifted Indicator and Holographic Stimulation of Blue Light-Sensitive Opsins

**Figure 5:**
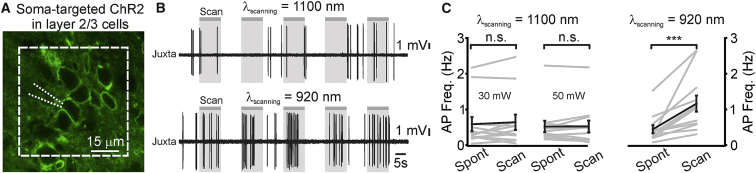
Scanning with Infrared-Shifted Wavelengths Does Not Modify the Activity of Cells Expressing Blue Light-Sensitive Opsins (A) Two-photon image of layer 2/3 cells expressing the soma-targeted ChR2-eYFP (green) *in vivo*. One ChR2^+^ neuron was recorded with a glass pipette (dotted white lines) while two-photon raster scanning inside the indicated area (dashed white line) was performed at 11 Hz. (B) Traces recorded in the juxtasomal configuration from one soma-targeted ChR2-expressing neuron during epochs (gray bars) of two-photon raster scanning at wavelength 1,100 nm (top) and wavelength 920 nm (bottom). Laser power: 30 mW in both conditions. (C) Average AP frequency during epochs of spontaneous activity (Spont) and during raster scanning (Scan). Left: results when scanning was performed at λ = 1,100 nm (laser power: 30 and 50 mW). Right: results when scanning was done at λ = 920 nm (laser power: 30 mW). Frame rate: 11 Hz; scanned area: ∼60 × 60 μm^2^ for all experimental conditions. p = 0.47 for λ = 1,100 nm and 30 mW, p = 0.85 for λ = 1,100 nm and 50 mW, p = 5E−4 for λ = 920 nm and 30 mW, Wilcoxon signed rank test, N = 12 FOVs from 3 mice. In this figure, the black line represents the average and SEM, individual experiments are depicted in gray.

Red-shifted channelrhodopsins have been used for two-photon stimulation of single neurons simultaneously with genetically encoded calcium indicators (GECI)-based calcium imaging (Carrillo-Reid et al., 2016, Packer et al., 2015, Rickgauer et al., 2014). One potential drawback of this approach is the remaining absorption by all red-shifted channelrhodopsins in the blue range of their action spectrum (Figure S1) (Mattis et al., 2011). As shown earlier, our data demonstrate that holographic illumination of blue light-sensitive opsins (ChR2 and GtACR2) at λ = 920 nm can be used to bidirectionally control the excitability of cortical neurons with high spatial resolution *in vivo*. We therefore asked whether this stimulation approach could be coupled with imaging of red-shifted functional indicators (e.g., jRCaMP), which are typically best excited in the two-photon regime at longer wavelength (λ = ∼1,100 nm) (Dana et al., 2016, Dunn et al., 2016), a wavelength at which blue light-sensitive channelrhodopsins show no detectable activity (Figure S1) (Prakash et al., 2012). To test this possibility, we first evaluated whether raster scanning at these long wavelengths caused opsin activation, leading to significant alteration of neuronal spiking activity *in vivo*. We expressed the soma-targeted ChR2 in layer 2/3 cortical neurons and performed juxtasomal electrophysiological recordings from ChR2^+^ neurons while raster scanning the field of view (FOV) containing the recorded cell at λ = 1,100 nm and scan rate of 11 Hz (scan resolution: 0.58 μm/pixel; dwell time: 4 μs) (Figure 5). We found that raster scanning did not significantly affect the firing activity of layer 2/3 soma-targeted ChR2^+^ neurons at both 30 and 50 mW imaging power (Figures 5B and 5C, left). As an important control, we found that raster scanning the same FOV at shorter wavelength (λ = 920 nm) and moderate power (laser power: 30 mW) increased the spiking activity of soma-targeted ChR2-expressing neurons (Figure 5C, right). Moreover, whole-cell, current-clamp recordings performed on layer 2/3 neurons expressing the inhibitory opsin GtACR2 showed that raster scanning at λ = 1,100 nm did not significantly modify the average resting membrane potential of opsin-positive neurons (average membrane potential: −62.1 ± 3.3 and −62.1 ± 3.3 mV in the absence and presence of raster scanning [laser power: 30 mW], respectively; −63.7 ± 3.2 and −63.9 ± 3.6 mV in the absence and presence of raster scanning [laser power: 50 mW], respectively; p = 1, Wilcoxon signed rank test at both 30 and 50 mW, N = 4 from 2 mice), as expected from the two-photon absorption spectrum of GtACR2 (Figure 4C).

**Figure 6:**
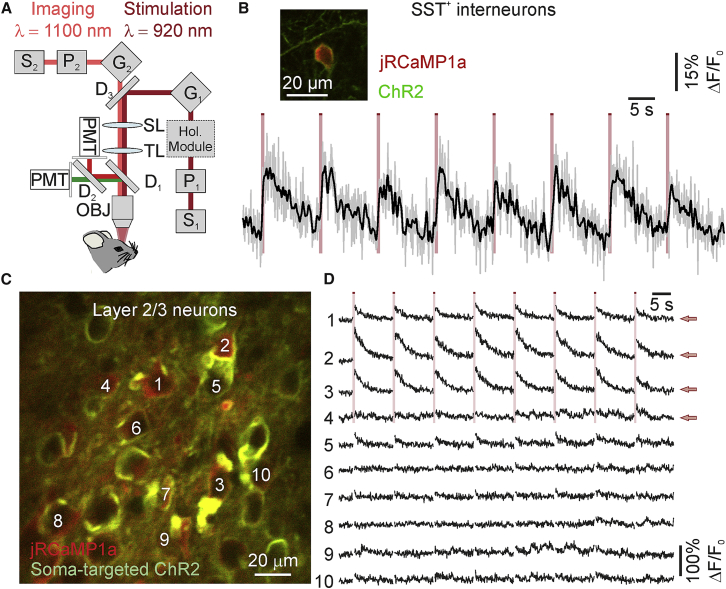
Simultaneous Two-Photon Imaging of Red-Shifted Indicator and Two-Photon Holographic Stimulation of Blue-Shifted Excitatory Opsin *In Vivo* (A) Schematic of the optical setup for simultaneous two-photon imaging (λ_exc_ = 1,100 nm) and two-photon holographic illumination (λ_exc_ = 920 nm). S_1_, stimulation laser source; S_2_, imaging laser source; P_1–2_, Pockels cells; G_1–2_, galvanometric mirrors; SL, scan lens; TL, tube lens; D_1–3_, dichroic mirrors; PMT, photomultiplier tube; OBJ, objective; Hol. Module, holographic module (comprising the SLM, the λ_1/2_, and L_1–4_ displayed in Figure 1). (B) Calcium transients in a SST^+^ interneuron during simultaneous two-photon imaging (λ = 1,100 nm; laser power: 25 mW; frame rate: 11 Hz; scanned area: ∼90 × 90 μm^2^) and holographic stimulation (λ = 920 nm; laser power: 50 mW). ΔF/F_0_ (gray trace) was smoothed with a moving average filter (black trace). The inset shows one layer 2/3 SST^+^ interneuron co-expressing ChR2-eYFP (green) and jRCaMP1a (red). (C) Two-photon image showing layer 2/3 neurons expressing soma-targeted ChR2-eYFP (green) and jRCaMP1a (red) *in vivo*. (D) Calcium transients recorded from jRCaMP1a-positive cells (imaging power: 30 mW; frame rate: 11 Hz). The numbers on the left refer to the neurons indicated in (B). Neurons 1–4 (red arrows) were simultaneously stimulated with four elliptical shapes covering the cell somata. Each stimulation episode is indicated by a red bar (stimulation power *per* cell: ∼50 mW). Periods of stimulation are blanked (see Experimental Procedures). See also Figures S6 and S7.

We combined holographic stimulation with raster scanning imaging to perform simultaneous two-photon imaging of jRCaMP1a and two-photon activation of ChR2 *in vivo*. Using two laser sources (Figure 6A) tuned at 1,100 nm (for imaging) and 920 nm (for holographic stimulation), we performed concurrent imaging and holographic photostimulation experiments in mice that co-expressed jRCaMP1a and soma-targeted ChR2 in layer 2/3 cortical neurons (Figure 6) or jRCaMP1a and ChR2 in SST^+^ interneurons (Figure 6B). We found that successive holographic stimulation of the cell body of ChR2-expressing neurons (stimulus power: 50 mW/cell) reliably evoked fluorescent transients in the stimulated cell (Figures 6B–6D). Combined juxtasomal electrophysiological and imaging recordings from jRCaMP1a-expressing neurons *in vivo* (Figure S6) confirmed that we could detect single and trains of APs with good accuracy (Dana et al., 2016). We combined this all-optical approach with the photostimulation of multiple specified neurons. We controlled the SLM to generate extended shapes covering the cell bodies of a group of four neurons (Figures 6C and 6D). We photostimulated the selected neurons (stimulus power: 56 mW/cell) while simultaneously imaging these and the surrounding neurons at 11 Hz. Targeted neurons that displayed clear ChR2-expression (Figures 6C and 6D, neuron 1–3) showed strong and reliable responses to photostimulation. Neighboring neurons responded weakly to holographic stimulation of target neurons, as expected from previous work (Packer et al., 2015).

Moreover, in cells expressing only jRCaMP1a, we controlled for potential artifacts induced by holographic stimulation on jRCaMP1a fluorescence. We recorded jRCaMP1a signals at 1,100 nm while performing repetitive short (Figures S7A and S7B) or prolonged (Figures S7C and S7D) holographic stimulation at 920 nm. We found that stimulation of the imaged cell (stimulation power: 30 mW) generated an artifact in the jRCaMP1a signal that could be removed by background subtraction. Increasing stimulation power from 30 to 50 mW resulted in similar effects (Figure S7E). Repetitive stimulation did not decrease jRCaMP1a baseline, and it did not induce evident signs of jRCaMP1a photobleaching (Figures S7F–S7H). Similarly, in background-subtracted traces, photostimulation did not significantly affect the amplitude and the off kinetics of the responses to whisker deflection (Figures S7I–S7M).

**Figure 7:**
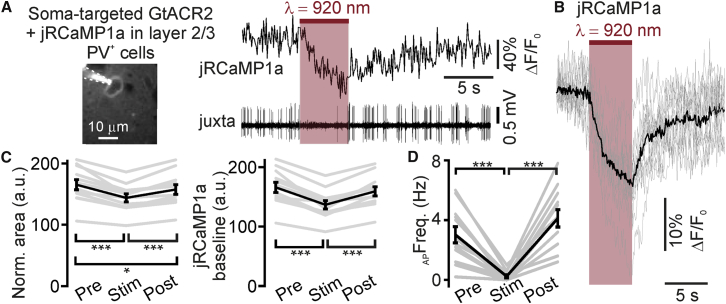
Simultaneous Two-Photon Imaging and Two-Photon Holographic Inhibition *In Vivo* (A) Left: image of a layer 2/3 PV^+^ interneuron co-expressing jRCaMP1a and the soma-targeted GtACR2 *in vivo*. The neuron was imaged and simultaneously recorded in the juxtasomal configuration. The patch pipette containing Alexa 594 is indicated by the dashed lines. Right: trace showing jRCaMP1a fluorescence (top, excitation wavelength: λ = 1,100 nm) and the spiking activity (bottom) for the neuron shown in (A). The cell was illuminated with a two-photon elliptical shape (red bar, λ = 920 nm; stimulation power: 50 mW; stimulus duration: 5 s). (B) Average decrease (ΔF/F_0_) (black trace) in jRCaMP1a fluorescence induced by holographic illumination at λ = 920 nm (pink bar) in cells co-expressing jRCaMP1a and the soma-targeted GtACR2. The gray traces represent single experiments. N = 12 cells from 3 mice. Imaging power range: 20–34 mW; stimulation power: 50 mW. (C) Area below the fluorescence trace (left) and fluorescence baseline level (middle) Pre, during (Stim), and Post holographic illumination (excitation wavelength: λ = 920 nm; stimulation power: 50 mW) in cells co-expressing jRCaMP1a and the soma-targeted GtACR2. Average area: p = 4E−7, ANOVA test with Bonferroni correction, N = 12 cells from 3 mice; average fluorescence: p = 1E−8, ANOVA test with Bonferroni correction, N = 12 from 3 mice. (D) Firing frequency of PV^+^ interneurons Pre, during (Stim), and Post holographic illumination. Excitation wavelength: λ = 920 nm; stimulation power: 50 mW; stimulus duration: 5 s. p = 8E−6, ANOVA test with Bonferroni correction, N = 12 stimulation trials from 3 neurons. In this figure, the black line represents the average and SEM, individual experiments are depicted in gray.

Finally, we performed simultaneous two-photon imaging and patterned photoinhibition in PV^+^ cells co-expressing jRCaMP1a and the soma-targeted GtACR2 (Mahn et al., 2017) *in vivo*. These neurons display a high spontaneous firing rate under our experimental conditions (Figures 3A, 3C, 7A, and 7D). We found that patterned illumination decreased the baseline jRCaMP1a signal in the stimulated cell (Figures 7A–7C). Simultaneous electrophysiological recording of the stimulated neuron confirmed that the baseline reduction in jRCaMP1a signal was associated with a decrease in the spike rate of the stimulated neuron (Figures 7A and 7D).

## Discussion

Simultaneous two-photon imaging and manipulation is increasingly recognized as a crucial tool for the causal investigation of brain networks (Bovetti and Fellin, 2015, Carrillo-Reid et al., 2017, Emiliani et al., 2015, Grosenick et al., 2015). Such a technique allows perturbing the activity of functionally identified ensembles of neurons and testing of the role of specific activity patterns in the regulation of network dynamics and behavior (Carrillo-Reid et al., 2017, Dal Maschio et al., 2017, Panzeri et al., 2017). Here we developed an all-optical approach for simultaneous two-photon imaging of a red-shifted functional indicator and bidirectional perturbation of neural activity using blue light-sensitive opsins *in vivo*. We validated our approach across different cell types and layers of the mouse neocortex. This is a fundamental step to apply all-optical methods to investigate the role of precise spatiotemporal activity patterns in driving higher cortical functions, because activity patterns are distributed in space and time across cellular subtypes (Carrillo-Reid et al., 2017).

Our method expands the potential of simultaneous imaging and perturbation for the functional dissection of brain circuits. Previous work in the mammalian brain *in vivo* (Carrillo-Reid et al., 2017, Packer et al., 2015, Rickgauer et al., 2014) demonstrated that the blue light-sensitive calcium indicator GCaMP (Chen et al., 2013, Tian et al., 2009) can be coupled to the red-shifted excitatory opsin C1V1 (Yizhar et al., 2011) for simultaneous two-photon imaging and perturbation (see also Supplemental Information). However, red-shifted opsins generally display a blue-shifted tail in their absorption spectrum that may complicate spectral separation and lead to crosstalk between GCaMP imaging and opsin activation under certain conditions (Packer et al., 2015). This is especially true for red-shifted excitatory opsins with long off kinetics that are often the preferred choice for two-photon activation using scanning approaches (Prakash et al., 2012). We showed (Figures 5, 6, and 7) that the use of jRCaMP1a, a red-shifted functional indicator that is excited using two-photon stimulation between 1,050 and 1,150 nm, in combination with blue light-sensitive opsins (e.g., ChR2) that display maximal two-photon excitability around 920 nm, minimizes this form of crosstalk. Using combined imaging and electrophysiological recordings (Figure 5), we found that the activity of ChR2-expressing cells was not changed by raster scanning *in vivo* (λ_imaging_ = 1,100 nm; laser intensity: 30–50 mW; frame rate: 11 Hz; scan resolution: 0.58 μm/pixel; FOV dimension: 58 × 58 μm^2^), in agreement with what observed in cultured neurons (Figure S1). The absence of crosstalk likely stems not only from the spectral separation of the two light-sensitive molecules that we have used (i.e., jRCaMP1a and ChR2) but also from the fast closing kinetics of ChR2 (Lin et al., 2009) and the low power required for imaging. The experimental configuration that we presented provides other advantages. For example, the use of red-shifted indicators may facilitate deep imaging by using longer wavelengths for fluorescence excitation and emission, which are less sensitive to tissue scattering (Helmchen and Denk, 2005). In addition, because of their stability for long-term expression (Dana et al., 2016), they can be efficiently used for chronic experiments. Moreover, the stimulation of blue light-sensitive opsins at 920 nm may decrease tissue heating that is higher at the longer wavelengths (λ = 1,040 nm) (Podgorski and Ranganathan, 2016) used to stimulate red-shifted opsins (e.g., C1V1) (Carrillo-Reid et al., 2017, Packer et al., 2012, Packer et al., 2015, Prakash et al., 2012, Rickgauer et al., 2014). Although photocurrents generated by ChR2 are generally smaller than those generated by C1V1 (Klapoetke et al., 2014, Yizhar et al., 2011), the two-photon cross section of ChR2 is high (Rickgauer and Tank, 2009), and cells responded efficiently to stimulation (Figures 1, 2, and 3).

Previous work *in vivo* demonstrated high spatial resolution two-photon activation of excitatory opsins (Carrillo-Reid et al., 2017, Packer et al., 2015, Rickgauer et al., 2014). Here we show that holographic two-photon illumination can be used for efficient suppression of neural activity with high spatial resolution and can be coupled with functional imaging for all-optical readout and inhibitory optogenetic manipulation *in vivo*. Although previous evidence *in vitro* showed that some light-sensitive proton pumps are excitable with a two-photon process (Prakash et al., 2012), here we focused on the use of chloride-permeable anion channelrhodopsins. We reasoned that the increased flow of ions *per* photocycle that characterizes light-sensitive channels would allow generation of larger photocurrents and more efficient hyperpolarization of neurons *in vivo* compared to the use of light-sensitive pumps. Among the various chloride-permeable opsins (Berndt et al., 2014, Wiegert et al., 2017, Wietek et al., 2014), we focused on GtACR2 because of its higher single-channel conductance and its blue light-sensitive, single-photon absorption spectrum (Govorunova et al., 2015). We first demonstrated that GtACR2 was efficiently stimulated through an absorption process that is compatible with two-photon excitation. Significant photocurrents were generated through holographic illumination of the cell body of GtACR2-expressing neurons in brain slice preparation (Figures 4A and 4B). The photocurrent had maximal peak amplitude for λ = 920 nm, similar to ChR2 (Mohanty et al., 2008, Rickgauer and Tank, 2009). Moreover, holographic stimulation of GtACR2 significantly hyperpolarized principal neurons *in vivo* and efficiently reduced their firing rate while maintaining high spatial resolution of the optogenetic perturbation (Figures 4D–4F). Most importantly, holographic stimulation of GtACR2 could be efficiently coupled with jRCaMP1a imaging for simultaneous functional imaging and optogenetic inhibitory manipulation with high spatial resolution (Figure 7). We observed a decrease in the baseline jRCaMP1a fluorescence in PV^+^ cells expressing GtACR2 upon patterned illumination at 920 nm (Figures 7A and 7B). Activation of GtACRs may change the intracellular chloride concentration and may lead to pH variations. These modifications might interfere with the fluorescence activity reporter. However, two lines of evidence suggest that the decrease in jRCaMP1a baseline activity upon patterned stimulation of GtACR2 is mainly due to a decrease in the cell’s firing rate. First, in simultaneous imaging and electrophysiological recordings, the baseline fluorescence decrease of jRCaMP1a was always associated with the decrease in the cell’s spiking rate (Figures 7A and 7D). Second, our observation is consistent with the high spontaneous firing rate of PV^+^ cells (Figure 3) being integrated by the slow activity reporter jRCaMP1a and with previous reports (Kato et al., 2015) showing decreased baseline of the fluorescence reporter upon sensory stimulation in PV^+^ interneurons corresponding to inhibited activity of these cells. A long recovery tail toward baseline level of the fluorescence reporter similar to the one observed in our experiments (Figures 7A and 7B) was also reported in that study (Kato et al., 2015). These results demonstrate that patterned two-photon optogenetics can be applied for high spatial precision optical inhibition of brain networks *in vivo*, making it possible to silence endogenous activity patterns triggered by sensory stimulation with very high cellular specificity.

Although our method efficiently decreased crosstalk between the imaging laser and the opsin activation, stimulation with extended shapes induced artifacts in the fluorescence detection, as observed by previous investigators (Baker et al., 2016). This artifactual signal may be due to unwanted stimulation by holographic illumination of the fluorescence protein that is tagged to the opsin (e.g., eGFP), the fluorescence of which may leak into the red fluorescence detection channel despite the barrier filter positioned in front of the photomultiplier tube (PMT). Alternatively, because jRCaMP1a has low, but not negligible, absorption at 920 nm (i.e., the wavelength used for stimulation), the artifactual signal may originate from direct activation of jRCaMP1a by holographic stimulation (Figure S7). If jRCaMP1a is expressed at high levels and the area covered by stimulated neuronal somata represents a significant portion of the FOV (e.g., when many neurons are stimulated at the same time), the integrated emission of dim jRCaMP1a fluorescence generated by holographic stimulation at 920 nm may generate significant artifacts in the red detection channel, as suggested by our experiments (Figure S7). This artifactual signal could be removed using background subtraction (Figure 6B; Figure S7) or required a blanking period (Figure 6D). A solution to this problem could be to synchronize photostimulation with imaging so that stimulation is performed when the portions of the FOV that are of no interest are being scanned (Baker et al., 2016). For stimulus duration longer than frame duration, optimization of protein expression levels or further developments in red-shifted indicators with reduced absorption at the wavelength used for patterned illumination will be needed.

In conclusion, we provide an experimental approach to image and bidirectionally manipulate brain networks with high spatial resolution in living animals. This all-optical approach will likely represent a powerful tool to dissect how activity patterns in specified ensembles of neurons determine brain function and animal behavior.

## Experimental Procedures

### Animal Surgery

All experiments were carried out according to the guidelines of the European Communities Council Directive and approved by the Instituto Italiano di Tecnologia (IIT) Animal Health Regulatory Committee and by the National Council on Animal Care of the Italian Ministry of Health (authorization 29-2011-A, 34/2015-PR). Animals were housed in individually ventilated cages under a 12-hr light:dark cycle. A maximum of 5 animals *per* cage was allowed. Access to food and water was *ad libitum*. Experiments were performed on young-adult animals (5–16 weeks old for *in vivo* experiments, 4–7 weeks old for *in vitro* experiments, either sex). Details about animal strains and viral injections are described in the Supplemental Experimental Procedures. For *in vivo* experiments, mice were anesthetized with intraperitoneal urethane (16.5%, 1.65 g/kg). The scalp was removed while infiltrating all incisions with lidocaine. A chamber with a central hole (hole diameter: 4 mm) was attached with dental cement to the animal’s skull for head-fixation. A craniotomy (∼700 × 700 μm^2^) was opened over the somatosensory (or visual cortex, in the case of experiments in Scnn mice) cortex, and the dura was carefully removed (unless otherwise stated). The location of the craniotomy was guided by the intensity of the fluorescence signal of the expressed transgene. The surface of the brain was kept moist with normal HEPES-buffered artificial cerebrospinal fluid (ACSF) (composed of 127 mM NaCl, 3.2 mM KCl, 2 mM CaCl_2_, and 10 mM HEPES [pH 7.4]). Body temperature was maintained at 37°C with a heating pad. Respiration rate, heartbeat, eyelid reflex, vibrissae movements, and reactions to tail pinching were typically monitored throughout the surgery and the experiment.

### Optical Setup and Phase Modulation for Holographic Illumination

See Supplemental Experimental Procedures.

### *In Vivo* Electrophysiological Recordings

Two-photon targeted juxtasomal electrophysiological recordings were performed as described in De Stasi et al. (2016) and Zucca et al. (2017). Borosilicate glass pipettes were pulled with a resistance of 4–9 MΩ and were filled with ACSF solution mixed with Alexa Fluor 488 or 594 (20 μM). Neurons were targeted by imaging the fluorescent reporter with the two-photon microscope while monitoring the pipette electrical resistance by applying brief voltage pulses. Additional details are reported in the Supplemental Experimental Procedures.

### *In Vivo* Two-Photon Imaging and Photostimulation

Two-photon imaging (λ_exc_ = 1,050 nm) was performed to assess the expression pattern of the opsin (ChR2-eYFP or ChR2-eYFP soma targeted) and the calcium indicator (jRCaMP1a) at the same time. A reference image of the selected FOV was acquired, and shapes covering the soma of target neurons were generated by the SLM (λ_exc_ = 920 nm) and projected at the sample. Temporal series were acquired in raster scanning configuration with the imaging beam (100 × 100 pixels; frame rate: 11 Hz; pixel dwell time: 4 μs; λ_exc_ = 1,100 nm). Holographic photostimulation duration was 500 ms and was repeated at 0.08 Hz for 7–9 repetitions. For analysis, temporal series acquired *in vivo* were imported into the ImageJ/Fiji software to identify regions of interest (ROIs). For each ROI, the change in fluorescence relative to the baseline (ΔF/F_0_) was computed as a function of time with the fluorescence baseline (F_0_) calculated in ten frames at the beginning of the recorded session. Artifactual fluorescence signals due to holographic stimulation were removed by background subtraction (Figure 6B; Figure S7) or required blanking (Figure 6D).

### Slice Electrophysiology

See Supplemental Experimental Procedures.

### Data Analysis and Statistics

For juxtasomal recordings, traces were high-pass filtered (cutoff frequency: 10 Hz) and spikes were detected with a threshold criterion. The threshold value was adjusted for each recorded sweep and set >3 times the SD of the trace. For experiments in Figures 1, 6, and 3 and in Figures S3 and S5, AP firing frequency was calculated in a time window Pre (window duration: 1 s), Stim (duration: 0.5 s) and Post (duration: 1.5 s) holographic stimulation over 15–20 stimulation trials. Δ_AP_Freq was calculated as the difference between the firing frequencies of the Stim and Pre time windows. Opsin-positive cells (for definition, see *In vivo* electrophysiological recordings in the Supplemental Experimental Procedures) were considered responsive to holographic stimulation when Δ_AP_Freq was >1 times the firing frequency in the Pre period at stimulation power ≤ 92 mW *per* shape. The fraction of opsin-positive neurons responding to holographic illumination was 14/16 for Ca^2+^/calmodulin-dependent protein kinase II-positive (CaMKII^+^) cells expressing ChR2 (Figures 1C–1E), 15/17 for layer 2/3 cells expressing ChR2 under the human synapsin promoter (Figures 1E and 1F), 31/33 for SST^+^ cells expressing ChR2 (Figure 3), 25/26 for PV^+^ cells expressing ChR2 (Figure 3), 19/20 for Scnn^+^ cells expressing ChR2 (Figure 3), and 21/21 for cells expressing the soma-targeted ChR2 under the human synapsin promoter (Figure 2). To compute the spatial resolution, neuronal responses (quantified as Δ_AP_Freq) were recorded first with the stimulation shape centered on the cell body and then during successive shifts of the excitation volume in the radial (20 μm steps) and in the axial (±25 μm steps) directions. Δ_AP_Freq as a function of the shift was then plotted for every recorded neuron in the three conditions (radial, axial_up_, and axial_down_) and fitted with a mono-exponential function (Δ_AP_Freq(x) = A ^∗^ exp(−l ^∗^ x)) (Packer et al., 2015). Fitting curves with l < 0 or with values of A that were different by more than 25% compared to Δ_AP_Freq at position x = 0 were not considered. The spatial resolution, l_1/2_, was defined as the distance at which the evoked response (calculated from fit) was equal to A/2. For the analysis of the recordings from GtACR2 expressing neurons, see Supplemental Experimental Procedures.

### Statistical Methods

All values are expressed as mean ± SEM unless otherwise stated. For each experimental group, sample size was chosen based on previous studies (Carrillo-Reid et al., 2016, Packer et al., 2015, Rickgauer et al., 2014). No statistical methods were used to predetermine sample size. All recordings with no technical issues were included in the analysis. For N ≥ 10, a Kolmogorov-Smirnov normality test was used to test for normality. For N < 10, a Saphiro-Wilk normality test was adopted. In case of normal distribution, Student’s t test was used to calculate statistical significance when comparing two populations of data. For non-normal distributions, the non-parametric Mann-Whitney test or Wilcoxon signed-rank test (for unpaired or paired comparison, respectively) was used unless otherwise stated. When multiple (>2) populations of data were compared, one-way ANOVA with Bonferroni or Tukey’s honestly significant difference (HSD) *post hoc* test was used in case of Gaussian distribution. For non-normal distribution and multiple comparisons, the non-parametric Friedman test with Dunn’s *post hoc* correction was used. All tests were two sided. Statistical analysis was performed using Prism (GraphPad, La Jolla, CA) and OriginPro 9.1 (OriginLab).

## Supplemental Information

Document S1. Supplemental Experimental Procedures, Figures S1–S7, and Tables S1–S3

Document S2. Article plus Supplemental Information
