# Supplementary material for: Two-Photon Bidirectional Control and Imaging of Neuronal Excitability with High Spatial Resolution In Vivo
Source: Cell Rep. 2018 Mar 13;22(11):3087–98. doi: 10.1016/j.celrep.2018.02.063 (PMC5863087; doi:10.1016/j.celrep.2018.02.063)
Supplement: Document S1. Supplemental Experimental Procedures, Figures S1–S7, and Tables S1–S3 [file mmc1.pdf]

Cell Reports, Volume *22*

Supplemental Information

Two-Photon Bidirectional Control and Imaging of Neuronal Excitability with High Spatial Resolution *In Vivo*

Angelo Forli, Dania Vecchia, Noemi Binini, Francesca Succol, Serena Bovetti, Claudio Moretti, Francesco Nespoli, Mathias Mahn, Christopher A. Baker, McLean M. Bolton, Ofer Yizhar, and Tommaso Fellin

Supplemental Figures

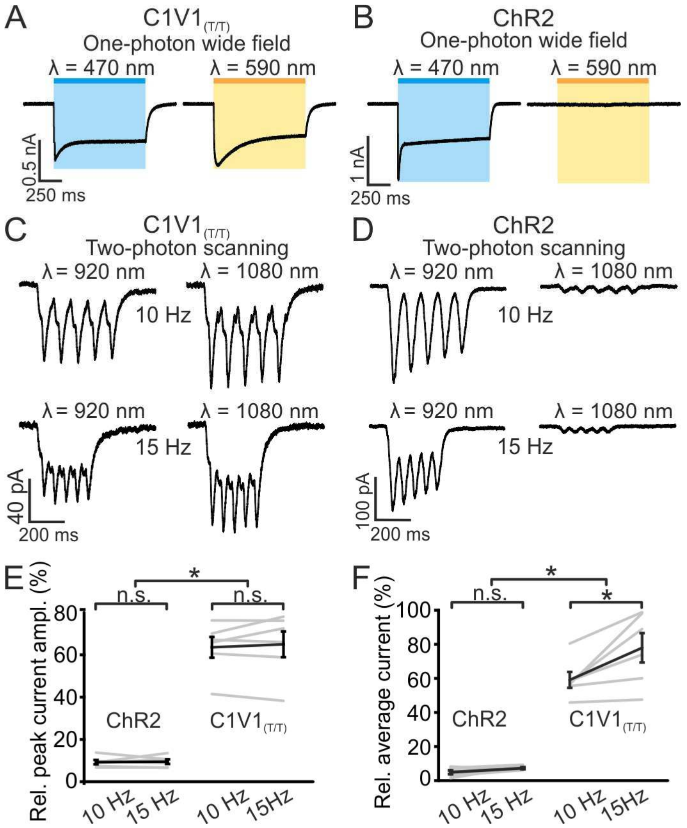

Figure S1. Two-photon raster-scanning induced cross-activation is significantly smaller for ChR2 than for C1V1. Related to Figure 1. **(A)** Representative traces of photocurrents induced by 470 nm and 590 nm one-photon (1P) wide-field illumination (stimulus duration: 1 s; stimulus intensity: 2 mW/ mm^2) in a cultured neuron expressing $C1V1_{(T/T)}$. Holding potential: -60 mV. **(B)** Same as in (A) for a neuron expressing ChR2. **(C)** Representative traces of photocurrents induced by raster-scanning (beam power: 25 mW) at the two-photon wavelengths typically used for calcium indicator imaging (920 nm and 1080 nm for green and red calcium indicators, respectively). A $C1V1_{(T/T)}$ expressing neuron was scanned at a frame rate of 10 Hz (top) and 15 Hz (bottom), using 920 nm (left) and 1080 nm (right). **(D)** Same as in (C) for a ChR2 expressing neuron. **(E)** Quantification of the peak photocurrent at the imaging wavelength (1080 nm and 920 nm for ChR2 and $C1V1_{(T/T)}$, respectively), normalized to the peak current of the 10 Hz scan at the activation wavelength (920 nm and 1080 nm for ChR2 and $C1V1_{(T/T)}$, respectively). This relative peak photocurrent was significantly higher for $C1V1_{(T/T)}$ expressing neurons than for ChR2 expressing neurons, p = 2E-6, ANOVA test with Tukey's correction, N = 6 neurons *per* opsin) with no difference between the scan rates (p = 0.48). **(F)** Quantification of the average photocurrent evoked at the imaging wavelength normalized to the average photocurrent of the 10 Hz scan at the activation wavelength. The relative average current was significantly higher for $C1V1_{(T/T)}$ (p = 2E-6 ANOVA test with Tukey's correction, N = 6 neurons *per* opsin) and increased significantly between 10 Hz and 15 Hz in $C1V1_{(T/T)}$ but not ChR2 expressing neurons (p = 0.95 and 9E-3, for ChR2 and $C1V1_{(T/T)}$, respectively).

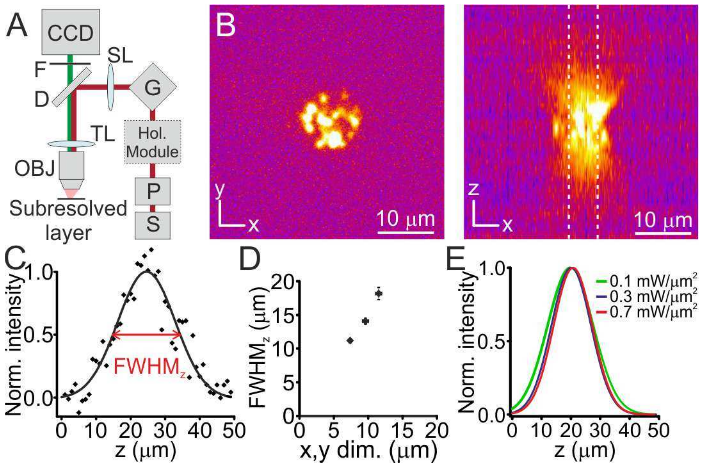

Figure S2. Axial profile of two-photon holographic illumination shapes. Related to Figure 1. **(A)** Optical setup used to characterize the axial profile of two-photon illumination shapes. S, laser source (λ = 920 nm); P, Pockels cell; Hol. module, holographic module; G, galvanometric mirrors; SL, TL, scan and tube lenses; D, dichroic mirror; OBJ, objective; F, fluorescence filter; CCD, CCD camera. **(B)** Top (left) and lateral (right) view of the fluorescence emission (in pseudo-color scale) generated by projecting an extended holographic shape (shape diameter, 10 µm) on an ultrathin fluorescent layer (Antonini et al., 2014). The z-stack is acquired by shifting the vertical position of the objective in 1 µm steps. **(C)** Normalized axial intensity profile for the z-stack shown in (B). Average fluorescence at a given axial position (single black points) was calculated inside circular regions (within the dashed lines indicated in the right panel in (B)) centered on the projected shape. Experimental values were fitted with a Gaussian curve (black line). **(D)** The axial full width at half maximum (FWHM$_z$) of projected shapes as a function of the lateral size of the illuminating shape (N = 5 projected shapes). **(E)** Normalized Gaussian fits obtained from the axial intensity profiles generated by projecting a holographic shape (shape diameter: 10 μm) at different power density values.

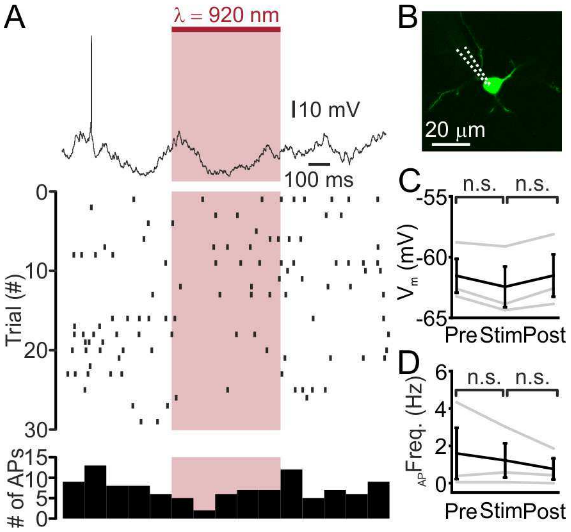

Figure S3. No effect of two-photon holographic illumination on opsin-negative neurons *in vivo*. Related to Figure 1. **(A)** Top: representative whole-cell current-clamp trace recorded before, during, and after single cell holographic stimulation (red bar, laser power: 80 mW; λ_{exc} = 920 nm) in an opsin negative layer 2/3 cortical neuron. Middle: raster plot showing cell response over consecutive trials for the same cell displayed on the top. Bottom: AP distribution for the trials shown in the middle panel (time bin: 100 ms). **(B)** Two-photon image of one opsin-negative layer 2/3 neuron which was recorded in whole-cell configuration and filled with Alexa Fluor 488 (green). Dotted lines indicate the glass pipette. **(C)** Average membrane potential before (Pre), during (Stim) and after (Post) holographic illumination of opsin-negative neurons in layer 2/3. p = 0.19, Friedmann test with Dunn's correction, N = 3 cells from 2 mice. Laser power: 80 mW; λ_{exc} = 920 nm. **(D)** Average firing frequency before (Pre), during (Stim) and after (Post) holographic illumination of opsin-negative neurons in layer 2/3. p = 0.53, Friedmann test with Dunn's correction, N = 3 cells from 2 mice.

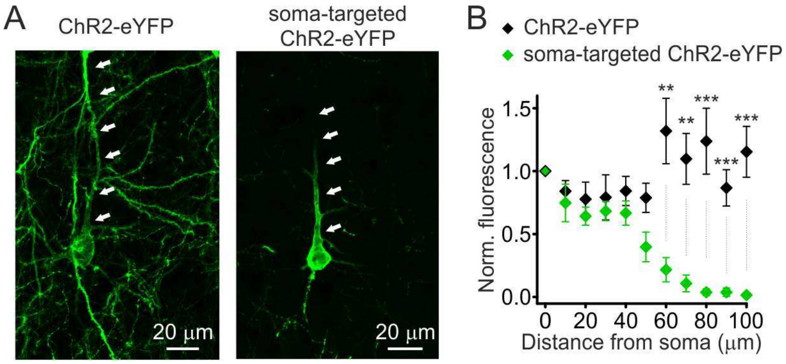

Figure S4. Opsin expression in neurites of cells expressing non soma-targeted ChR2 and soma-targeted ChR2. Related to Figure 2. **(A)** z-averaged confocal images of cortical neurons expressing non soma-targeted ChR2-eYFP (left) and soma-targeted ChR2-eYFP (right). Images are obtained from the projection of 10-20 single images acquired in subsequent planes (1 μm spacing). White arrows indicate the neurite along which fluorescence was measured. **(B)** Fluorescence intensity as a function of the position along a neurite. Fluorescence values are normalized to the fluorescence at the soma, for non soma-targeted ChR2-eYFP (N = 7 neurites from 7 cells from 3 mice) and for the soma-targeted ChR2-eYFP (N = 13 neurites from 13 cells from 3 mice). p = 4E-3, p = 2E-3, p = 3E-4, p = 3E-4, and p = 6E-5 for 60, 70, 80, 90, and 100 μm distance, respectively. Two-sample Kolmogorov-Smirnov test with Bonferroni correction.

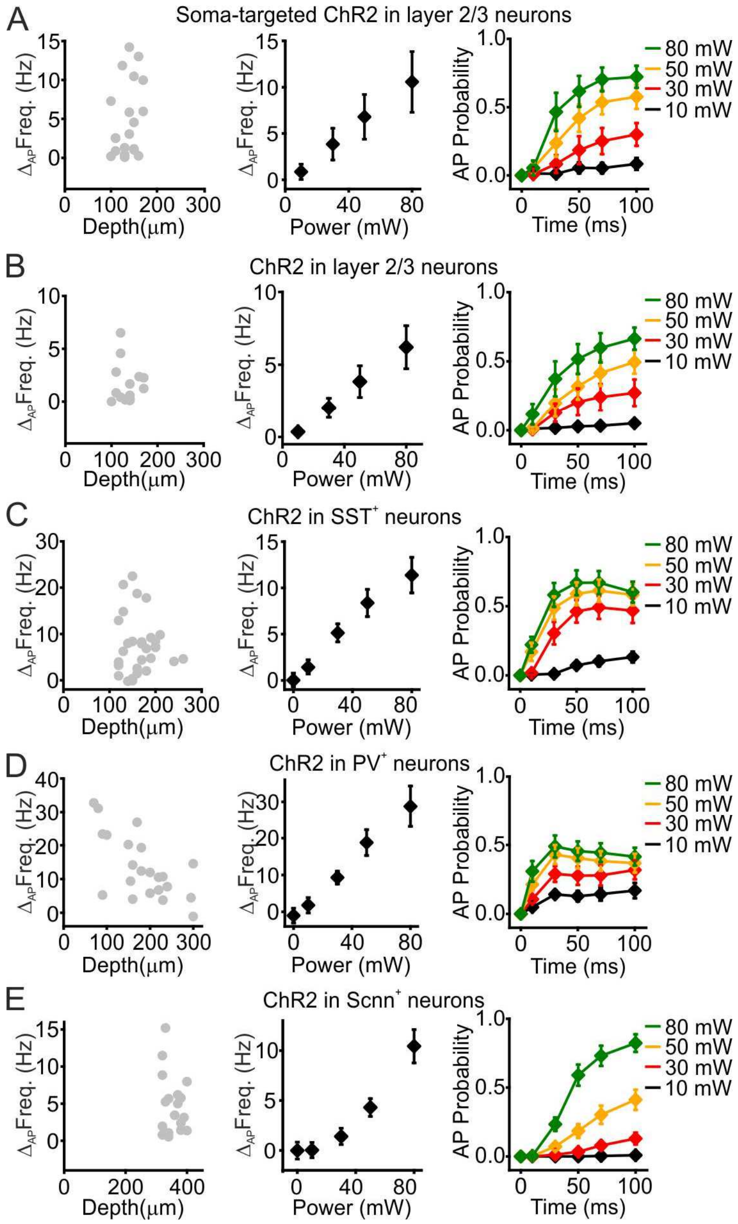

Figure S5. Firing probability during holographic illumination increases with stimulation power. Related to Figure 3. **(A)** Left panel: average AP frequency increase during holographic illumination (λ_{exc} = 920 nm) as a function of the depth of the recorded cells for layer 2/3 neurons expressing the soma-targeted ChR2. N = 21 neurons from 6 mice; laser power: 30 mW. Middle panel: average AP frequency increase during holographic illumination as a function of laser power. N = 8 cells from 4 mice. Right panel: average probability of firing during holographic illumination as a function of time after the beginning of the stimulation at different power levels. Data are corrected for the spontaneous firing rate of the recorded cell (See Methods). N = 8 cells from 4 mice. **(B)** Same as in (A) for layer 2/3 cells expressing ChR2. Left panel: N = 15 cells from 6 mice; laser power: 30 mW. Middle panel: N = 10 cells from 5 mice. Right panel: N = 10 cells from 5 mice. **(C)** Same as in (A) for layer 2/3 SST^+ cells expressing ChR2. Left panel: N = 31 cells from 7 mice; laser power: 30 mW. Middle panel: N = 12 cells from 3 mice. Right panel: N = 12 cells from 3 mice. **(D)** Same as in (A), but for layer 2/3 PV^+ interneurons expressing ChR2. Left panel: N = 22 cells from 7 mice, laser power: 30 mW. Middle panel: N = 13 cells from 5 mice. Right panel: N = 13 cells from 5 mice. **(E)** Same as in (A), but for layer 4 $Scnn^+$ neurons expressing ChR2. Left panel: N = 19 cells from 8 mice, laser power: 50 mW. Middle panel: N = 14 cells from 7 mice. Right panel: N = 14 cells from 7 mice.

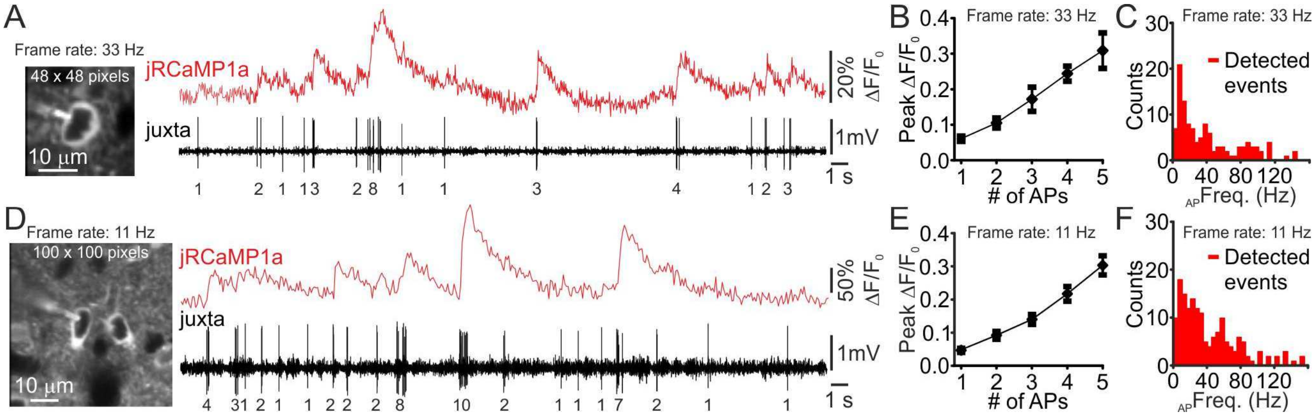

Figure S6. Sensitivity of jRCaMP1a imaging. Related to Figure 6. **(A)** Left: Two-photon image of a jRCaMP1a-expressing neuron which was imaged *in vivo* at 33 Hz frame rate with high zoom (40x objective, image dimension: 48 x 48 pixels) and simultaneously recorded in the juxtasomal electrophysiological configuration. The patch pipette containing Alexa 594 is visible on the left of the target neuron. Right: Simultaneous imaging (top, red trace) and juxtasomal electrophysiological recording (bottom, black trace) from a jRCaMP1a-expressing layer 2/3 neuron in an anesthetized mouse during spontaneous activity. The black numbers below the electrophysiological trace are the numbers of discharged APs. **(B)** Peak fluorescence change as a function of number of APs discharged by the recorded neuron (1AP, N = 162 events; 2AP, N = 70 events; 3AP, N = 31 events; 4AP, N = 26 events; 5AP, N = 18 events). Detection accuracies (see Methods) were: 1AP, 41%; 2AP, 70%; 3AP, 90%; 4AP, 100%; 5AP, 94% (N = 9 cells from 2 mice). **(C)** Distribution of average firing frequency during bursts of 2-5 APs for all detected events (red, 119/145 of total 2-5AP events). Bin dimension: 5 Hz. **(D)** Same as in (A) for a neuron recorded *in vivo* at frame rate of 11 Hz. **(E-F)** Same as (B-C) for jRCaMP1a signals acquired at 11 Hz. In (B) (1AP, 202 events; 2AP, 84 events; 3AP, 56 events; 4AP, 32 events; 5AP, 22 events). Detection accuracies: 1AP, 42%; 2AP, 74%; 3AP, 82%; 4AP, 97%; 5AP, 100% (N = 11 cells from 3 mice). In (F), detected events, 163/194 of total 2-5AP events.

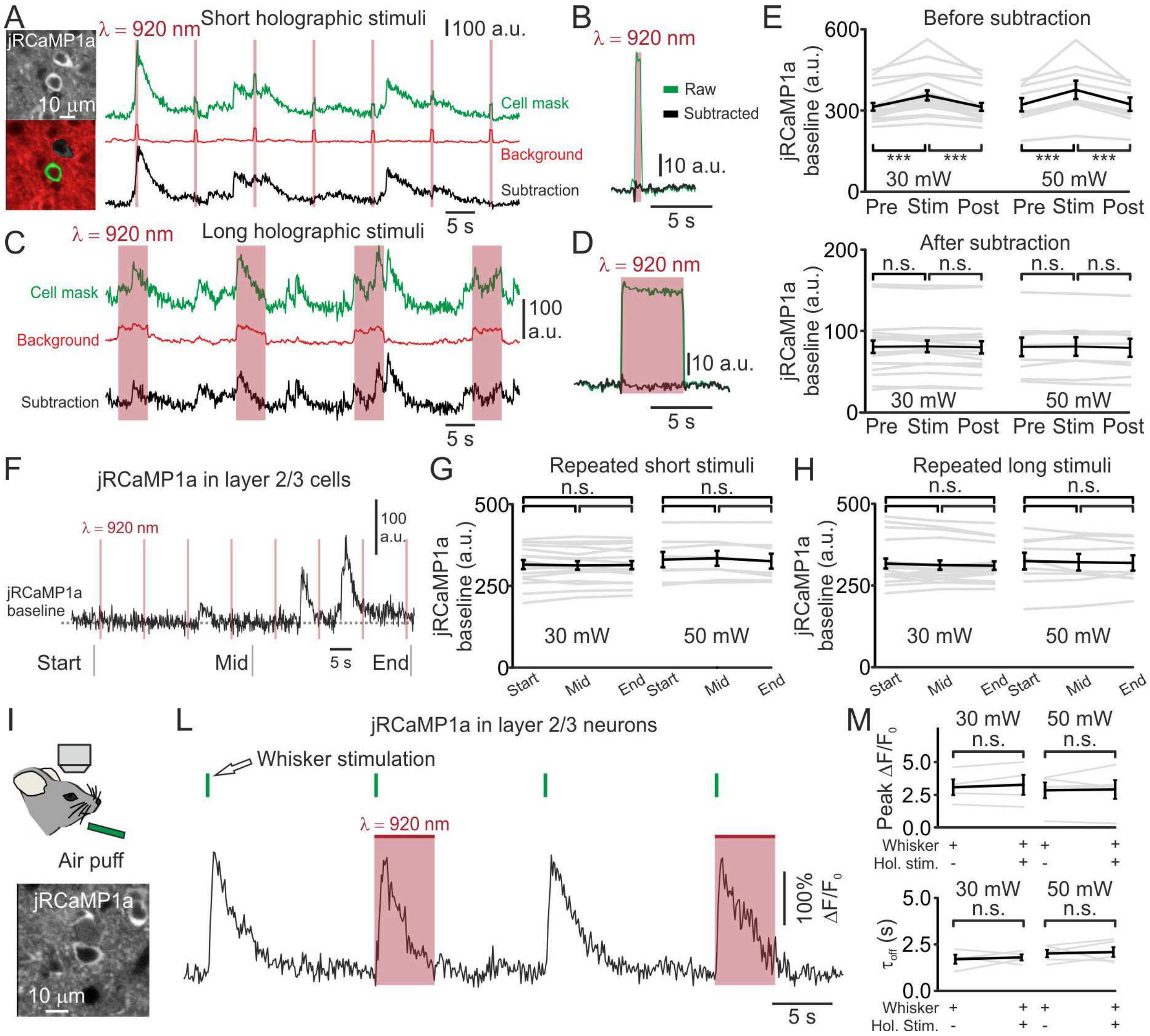

Figure S7. Holographic stimulation at λ = 920 nm generates small artifacts in jRCaMP1a fluorescence which can be effectively removed by subtraction. Related to Figure 6. **(A)** Top left: jRCaMP1a expressing layer 2/3 neurons imaged at λ = 1100 nm. Bottom left: somatic (green) and background (red) regions are identified. Right (from top to bottom): fluorescence signal in the somatic region (green, cell mask), background region (red, background), and estimated true fluorescence of the cell (black, subtraction) after background subtraction. Imaging wavelength: λ = 1100 nm. Holographic stimulation (red bar) was performed at λ = 920 nm. Laser power: 50 mW; stimulus duration: 500 ms. **(B)** Average detected fluorescence from jRCaMP1a-expressing neurons during brief holographic illumination. Raw data are shown in green, subtracted data in black (168 illumination periods from N = 21 neurons from 3 mice; stimulation laser power range: 30 - 50 mW; stimulus duration: 500 ms; 8 repetitions at 0.1 Hz *per* neuron; stimulation wavelength: λ = 920 nm; imaging laser power: 15 - 32 mW). **(C)** Same as in (A), but during holographic stimulation with longer pulses (pulse duration: 5 s). Stimulus power: 30 mW; stimulation wavelength: λ = 920 nm. **(D)** Same as in (B) for long holographic illumination periods (140 illumination periods from N = 28 neurons from 6 mice; stimulation laser power: 30 - 50 mW; stimulus duration: 5 s; 5 repetitions at 0.05 Hz *per* neuron; stimulation wavelength: λ = 920 nm; imaging laser power: 15 - 32 mW). The average value of fluorescence change induced by the artifact and expressed in $\Delta F/F_0$ was: 0.13 ± 0.02 (stimulation power: 30 mW, N = 19 from 6 mice) and 0.16 ± 0.03 (stimulation power: 50 mW, N = 9 from 4 mice). **(E)** Top: baseline jRCaMP fluorescence from raw data before (Pre), during (Stim), and after (Post) holographic illumination at λ = 920 nm. Stimulus power: 30 mW (left), p = 2E-9 ANOVA test with Bonferroni correction, N = 19 neurons from 6 mice and 50 mW (right), p = 2E-4 ANOVA test with Bonferroni correction, N = 9 neurons from 4 mice. Bottom: same data as above for background subtracted jRCaMP1a signals. Stimulus power: 30 mW (left), p = 0.31 ANOVA test with Bonferroni correction, N = 19 neurons from 6 mice and 50 mW (right), p = 0.38 ANOVA test, N = 9 neurons from 4 mice. Holographic stimulus duration: 5 s; 5 repetitions at 0.05 Hz; imaging laser power range: 15 - 32 mW. **(F)** jRCaMP1a fluorescence (black trace) from a representative neuron during repeated holographic stimulations (red bars, at λ = 920 nm). jRCaMP1a baseline was measured

outside the illumination periods at the beginning (grey vertical line, Start), in the middle (grey vertical line, Mid), and at the end (grey vertical line, End) of the stimulation protocol. **(G)** Baseline jRCaMP fluorescence measured during Start, Mid and End epochs for repeated holographic illumination with brief stimuli at λ = 920 nm (light stimulus duration: 500 ms; 8 repetitions at 0.1 Hz). Left: stimulation laser power: 30 mW (p = 0.77, ANOVA test with Bonferroni correction, N = 17 neurons from 6 mice). Right: stimulation laser power: 50 mW (p = 0.08, ANOVA test with Bonferroni correction, N = 8 neurons from 5 mice). **(H)** Same as (G), but for holographic illumination with longer pulses (light stimulus duration: 5 s, 5 repetitions at 0.05 Hz). Left: stimulation laser power: 30 mW (p = 0.14, ANOVA test with Bonferroni correction, N = 19 neurons from 6 mice). Right: stimulation laser power, 50 mW (p = 0.7, ANOVA test with Bonferroni correction, N = 9 neurons from 4 mice). **(I)** Top: schematic of the experimental configuration for two-photon imaging of jRCaMP1a responses in the barrel cortex of an anesthetized mouse during stimulation of the contra-lateral whiskers with an air puff. Bottom: a layer 2/3 jRCaMP1a-expressing neuron imaged at λ = 1100 nm. **(L)** Representative trace showing jRCaMP1a fluorescence transients evoked in a layer 2/3 neuron by whisker stimulation (green bars; whisker stimulus duration: 300 ms) with or without simultaneous holographic illumination at λ = 920 nm (red bar; laser power: 50 mW; light stimulus duration: 5 s). **(M)** Peak amplitude ($\Delta F/F_0$, top) and decay time constant (τ_{off}, bottom) of jRCaMP1a responses evoked by whisker stimulation in the absence and presence of holographic illumination. Peak $\Delta F/F_0$: 30 mW, p = 0.44, paired Student's *t*- test, N = 4 neurons from 2 mice; 50 mW, p = 1, Wilcoxon signed rank test, N = 5 neurons from 2 mice. τ_{off}: 30 mW, p = 0.74, paired Student's *t*- test, N = 4 neurons from 2 mice; 50 mW, p = 0.8, paired Student's *t*- test, N = 5 neurons from 2 mice.

Cell type	Promoter	Opsin	Diameter (µm)	Depth (µm)	Density (#10^3/mm^3)	$l_{1/2}$ rad. (µm)	$l_{1/2}$ ax. up (µm)	$l_{1/2}$ ax. down (µm)	$P_{density}$ (mW/$µm^2$)
layer 2/3	hsyn	ChR2-eYFP	11.9 ± 0.1	134 ± 6	161.8 ± 20.7	20 ± 6	32 ± 7	16 ± 4	0.09-0.72
layer 2/3	hsyn	stChR2-eYFP	12.8 ± 0.3	136 ± 5	141.6 ± 10.8	10 ± 2	13 ± 3	21 ± 3	0.08-0.62
SST	EF1a	ChR2-mCherry	10.5 ± 0.2	166 ± 6	5.7 ± 0.6	11 ± 2	19 ± 3	24 ± 3	0.12-0.92
PV	EF1a	ChR2-mCherry	11.1 ± 0.5	186 ± 15	7.0 ± 1.5	12 ± 2	26 ± 4	28 ± 4	0.10-0.83
layer 4	EF1a	ChR2-eYFP	10.4 ± 0.3	354 ± 7	95.2 ± 18.8	6 ± 2	30 ± 9	19 ± 3	0.12-0.94
layer 2/3	hsyn	GtACR2-eGFP	12.5 ± 0.2	131 ± 4	150.6 ± 9.3	11 ± 4	33 ± 4	29 ± 7	0.08-0.65

Table S1. Parameters of holographic stimulation across experimental conditions. Related to Figure 1-4. Average ± s.e.m. values of the cell diameter (diameter), the cortical depth of the stimulated neurons (depth), the density of opsin-expressing cells in the corresponding cortical area (density), the spatial constant corresponding to the half maximal response in the radial ($l_{1/2}$ rad.) and axial ($l_{1/2}$ ax. up and $l_{1/2}$ ax. down) directions, and the power density range (at the focal plane and measured without biological tissue) under the different conditions tested in this study. The first three columns on the left indicate the cellular population, the promoter, and the type of opsin used in this study. stChR2-eYFP: soma-targeted ChR2-eYFP.

Cell type	Promoter	Opsin	$l_{1/2}$ rad. (diameter units)	$l_{1/2}$ ax. up (diameter units)	$l_{1/2}$ ax. down (diameter units)	$V_{1/2}$ (μm^3)	Add. Opsin$^+$ neurons in $V_{1/2}$
layer 2/3	hsyn	ChR2-eYFP	1.68	2.69	1.34	4.02E+04	6
layer 2/3	hsyn	stChR2-eYFP	0.78	1.02	1.64	7.12E+03	0
SOM	EF1a	ChR2-mCherry	1.05	1.81	2.29	1.09E+04	0
PV	EF1a	ChR2-mCherry	1.08	2.34	2.52	1.63E+04	0
layer 4	EF1a	ChR2-eYFP	0.58	2.88	1.83	3.69E+03	0
layer 2/3	hsyn	GtACR2-eGFP	0.88	2.64	2.32	1.57E+04	1

Table S2. Spatial resolution of holographic stimulation. Related to Figure 1-4. Average ± s.e.m. values of the spatial constant corresponding to the half maximal response in the radial ($l_{1/2}$ rad.) and axial ($l_{1/2}$ ax. up and $l_{1/2}$ ax. down) directions normalized to the diameter of the stimulated cells. The volume of illumination of half activation ($V_{1/2}$, see Supplemental Methods) and the number of additional opsin-positive cells within $V_{1/2}$ besides the target cell are also shown. The first three columns on the left indicate the cellular population, the promoter, and the type of opsin used in this study.

	Firing rate pre (Hz)	Firing rate post (Hz)	p value
**Layer 2/3 ChR2**	0.60 ± 0.24	0.67 ± 0.30	1.00 (N = 10)
**Layer 2/3 stChR2**	0.98 ± 0.56	0.80 ± 0.43	0.55 (N = 8)
**Layer 4 ChR2**	0.07 ± 0.02	0.08 ± 0.02	0.86 (N = 14)

Table S3. Effect of holographic stimulation on the electrical properties of illuminated neurons. Related to Figure 1-3. Average ± s.e.m. values of the firing frequency of layer 2/3 and layer 4 neurons before (Pre) and after (post) holographic illumination. Stimulus duration: 500 ms; stimulus power: 80 mW. Wilcoxon signed rank test was used under all three conditions.

Supplemental Experimental Procedures

Animal Strains

C57BL/6J mice (Charles River, Calco, Italy), B6;129S6-*Gt(ROSA)26Sor*$^{tm14(CAG-TdTomato)Hze}$/J (JAX #007908 referred here as tdTomato line), B6;129P2-*Pvalb*$^{tm1(cre)Arbr}$/J (JAX #008069, called PV-cre line), STOCK*Sst*$^{tm2.1(cre)Zjh}$/J (JAX #013044, called SST-cre line), B6;C3-Tg(Scnn1a-cre)3Aibs/J (JAX #009613,called Scnn-cre line) were purchased from the Jackson Laboratory (Bar Harbor, USA) and used in this study.

Transgene expression

The adeno-associated viruses (AAVs) AAV1-EF1a-dFlox-hChR2(H134R)-mCherry-WPRE-hGH, AAV1-EF1a-DIO-hChR2(H134R)-eYFP-WPRE-hGH, AAV1.hsyn.ChR2(H134R).EYFP.WPRE, AAV1.CAG.flex.NES.jRCaMP1a, AAV9-EF1a-DIO-hChR2(H134R)-eYFP-WPRE-hGH, AAV1-CAG-flex-tdTomato-WPRE-hGH, AAV1-CamKII0.4-Cre-SV40, AAV1syn.NES.jRCaMP1a, AAV1syn.flex.NES.jRCaMP1a were purchased from the University of Pennsylvania Viral Vector Core. AAV carrying GtACR2 (AAV2/1.hSyn.GtACR2.eGFP and AAV2/1.hSyn1.SIO.stGtACR2-FRed.WPRE) was produced as previously described in (Mahn et al., 2016) and (Mahn et al., 2017), respectively. AAVs carrying ChR2 targeted to the soma (AAV1.hSyn.hChR2(H134R).eYFP.Kv2.1) was produced as in (Baker et al., 2016). Only opsins fused to fluorophore were used in this study. For direct comparison between ChR2 and soma-targeted ChR2 reported in the main text and supplementary figures, mice injected with AAV1.hsyn.ChR2(H134R).EYFP.WPRE or AAV1.hsyn.ChR2(H134R).EYFP.Kv2.1 were considered. Viral injections were performed on postnatal days 1 - 2 (P1 - P2; the day of birth was designated as P0) or in young adults (> P30) similarly to (Beltramo et al., 2013; Zucca et al., 2017). For the experiments in Figures 1-6B and Figure S4-5 in which injections were performed at P1 - P2, pups were deeply anaesthetized by placing them in ice for 4 minutes and then immobilized in a refrigerated custom stereotaxic apparatus. The skull above one brain hemisphere was exposed with a small incision to the skin and ~ 250 nl of viral suspension was slowly injected with a glass micropipette (stereotaxic coordinates: 1 mm posterior from bregma, 1.5 mm lateral of the sagittal sinus and at 0.25 mm depth). After the removal of the pipette, the skin was sutured and the pup was revitalized under an infrared heating lamp. 4 - 10 weeks after injection, mice were used for experiments. For the experiments displayed in Figure 1C-E, 4 - 7 weeks old animals were anesthetized with 2% isoflurane/0.8% oxygen, placed into a stereotaxic apparatus (Stoelting Co, Wood Dale, IL), and maintained on a warm platform at 37 °C for the whole duration of the anesthesia. After scalp incision, a small hole was drilled on the skull above the neocortex to lower the micropipette into the tissue (pipette depth: 0.25 - 0.3 mm from the pia). 1 µl of virus was injected at 30 - 50 nL/min by means of a hydraulic injection apparatus driven by a syringe pump (UltraMicroPump, WPI, Sarasota, FL). The scalp incision was then sutured and covered with antibiotic ointment, and the animals were monitored until recovery. 3 - 5 weeks after injection, mice were used for experiments. For combined imaging and photo-stimulation experiments (Figure 6C-D and Figure 7), injection of the AAV transducing the opsin and the AAV carrying the jRCaMP1a construct were performed in two subsequent injections with procedures similar to those described previously with the exception that for the AAV carrying the jRCaMP1a construct the injection was performed in 2 sites (200 nl injected solution in each site). Three-four weeks after this latter injection, mice were used for experiments. The same procedure except for the injection of the opsin was used for experiments in Figure S6 and S7.

Primary hippocampal neuron culture and transfection

Primary cultured hippocampal neurons were prepared from male and female P0 Sprague-Dawley rat pups (Envigo). CA1 and CA3 were isolated, digested with 0.4 mg/ml papain (Worthington), and plated onto glass coverslips pre-coated with 1:30 Matrigel (Corning). Cultured neurons were maintained in a 5% CO_2 humidified incubator with Neurobasal-A medium (Invitrogen) containing 1.25% fetal bovine serum (FBS, Biological Industries), 4% B-27 supplement (Gibco), 2 mM Glutamax (Gibco) and plated on coverslips in a 24-well plate at a density of 65,000 cells per well. To inhibit glial overgrowth, 200 µM fluorodeoxyuridine (FUDR, Sigma) was added after 4 days of in vitro culture (DIV). Neurons were transfected using the calcium phosphate method (PMID: 4705382). Briefly, the medium of primary hippocampal neurons cultured in a 24 well plate was collected and replaced with 400 µl serum-free MEM medium (ThermoFisher Scientific). 30 µl transfection mix (2 µg plasmid DNA and 250 µM $CaCl_2$ in HBS at pH 7.05) were added per well. After 1 h incubation the cells were washed twice with MEM and the medium was changed back to the collected original medium. Cultured neurons were used between 14 – 17 DIV for experiments. The plasmids pAAV-CaMKIIa-C1V1(T/T)-P2A-eYFP-WPRE and pAAV-CaMKIIa-ChR2(H134R)-eYFP-WPRE were used.

Electrophysiological recording in cultured neurons

Whole-cell patch-clamp recordings were performed under visual control using oblique illumination on a two-photon laser scanning microscope (Olympus 20x, 1.00 NA; Ultima IV, Bruker) equipped with a 12 bit monochrome CCD camera (QImaging QIClick-R-F-M-12). Borosilicate glass pipettes (Sutter Instrument BF100-58-10) with resistances ranging from 3 – 7 MΩ were pulled using a laser micropipette puller (Sutter Instrument Model P-2000). For hippocampal neuron

cultures, electrophysiological recordings from neurons were obtained in Tyrode's medium ([mM], 150 NaCl, 4 KCl, 2 $MgCl_2$, 2 $CaCl_2$, 10 D-glucose, 10 HEPES; 320 mOsm; pH adjusted to 7.35 with NaOH), AcOH Tyrode's medium ([mM], 125 NaCl, 25 AcOH, 4 KCl, 2 $MgCl_2$, 2 $CaCl_2$, 10 D-glucose, 10 HEPES; 320 mOsm; pH adjusted to 7.35 with NaOH) containing D-AP5 (25 µM; ab120003; Abcam) and CNQX (10 µM; C-141, Alomone). The recording chamber was perfused at 0.5 ml/min and maintained at 29°C. Pipettes were filled using standard intracellular solution ([mM], 135 K-gluconate, 4 KCl, 2 NaCl, 10 HEPES, 4 EGTA, 4 MgATP, 0.3 NaGTP; 280 mOsm/kg; pH adjusted to 7.3 with KOH). Whole-cell voltage clamp recordings were performed using a MultiClamp 700B amplifier, filtered at 8 kHz and digitized at 20 kHz using a Digidata 1440A digitizer (Molecular Devices).

In vitro illumination for cross-talk quantification
Whole-field illumination *in vitro* was performed using a 470 nm light emitting diode (LED; 29 nm bandwidth; M470L2-C2; Thorlabs) and 590 nm LED (18 nm bandwidth; M590L3-C2; Thorlabs) delivered through the microscope illumination path including a custom dichroic in order to reflect the activation wavelength. Light power densities were calculated by measuring the light transmitted through the objective using a power meter (Thorlabs PM100A with S146C sensor) and dividing by the illumination area, calculated from the microscope objective field number and magnification (PMID: 18974739). For quantification of two-photon imaging evoked opsin currents, a region of interest (ROI) of 100 µm x 100 µm was scanned using unidirectional raster-scanning with parameters typically used for calcium indicator imaging (4 µs dwell time, 100 x 100 pixel, 920 nm and 1080 nm at 25 mW average laser power).

Optical setup for _in vivo_ recordings
The optical set-up for two-photon holographic illumination was composed of two pulsed laser sources (S in Figure 1A and S_1 in Figure 6A, Chameleon Ultra II, 80 MHz repetition rate, tuned at 920 nm, and S_2 in Figure 6A Chameleon Discovery, 80 MHz repetition rate, tuned at 1100 nm, Coherent, Milan, IT), a customized scanhead (Bruker Corporation, former Prairie Technologies, Milan, IT), an upright epifluorescence microscope (BX61 Olympus, Milan, IT), and a liquid crystal spatial light modulator (SLM, X10468-07 SLM, Hamamatsu, Milan, IT). The laser beam intensity was modulated by a Pockels cell (P in Figure 1A and P_{1-2} in Figure 6A, Conoptics Inc, Danbury, CT) and then directed to the SLM by a sequence of mirrors (BB1-E03 Thorlabs, Newton, NJ). A half-wave plate (λ/2 in Figure 1A, RAC 5.2.10 achromatic λ/2 retarder - B. Halle Nachfl GMBH, Berlin, DE) was placed before the SLM in order to obtain the optimal polarization for phase-only modulation. A first telescope (L_1 and L_2 in Figure 1A, IR doublets 30 mm and 75 mm, Thorlabs, Newton, NJ) expanded the laser beam to fill the active window of the SLM. A second telescope (L_3 and L_4 in Figure 1A, IR doublets 300 mm and 150 mm, Thorlabs, Newton, NJ) was used to resize the laser beam to fit the dimensions of the scanning mirrors inside the scanhead (G in Figure 1A and G_1 in Figure 6A) and to optically conjugate the plane of the SLM with the back aperture of the objective. For alignment purposes, the SLM was mounted on a lab jack (L200/M, Thorlabs, Newton, NJ), a translator (PT1/M, Thorlabs, Newton, NJ), and a rotation platform (RP01/M, Thorlabs, Newton, NJ). Two multi-alkali photomultipliers tubes (PMTs, Hamamatsu, Milan, IT) were used as detectors for raster scanning imaging. Dual emission filters in front of the two PMTs were 525/70 nm and 607/45 nm, respectively. D_1 in Figure 1A and Figure 6A was a 660 nm long-pass dichroic mirror, D_2 a 575 nm long-pass dichroic mirror. The Olympus LUMPlanFl40X/IR objective (0.8 NA) was used for most experiments, except those in Figure 6, in which an Olympus XLPLN25XWMP2 (1.05 NA) was used. A mechanical shutter (Uniblitz, VCM-D1 Shutter Driver, Vincent Associates, Rochester, NY) controlled by a TTL signal was used to control holographic illumination duration. For the simultaneous two-photon imaging and stimulation experiments in Figure 6-7 and Figure S7, the telescope downstream the SLM was replaced by two IR doublets (400 mm and 125 mm, Thorlabs, Newton, NJ) and the stimulation beam was relayed onto a second set of galvanometric mirrors inside the scanhead (G_1 in Figure 6A). Imaging and stimulation beams were combined by a dichroic (D_3 in Figure 6A, zt980rdc, Chroma Technology Corporation, Bellows Falls, VT) positioned between the two sets of galvanometric mirrors and the scan lens. Calibration of the SLM projection plane at the sample and the imaging field of view (FOV) was performed by imaging the sample plane with a CCD camera (ORCA R2, Hamamatsu, Milan, Italy) *via* the objective and the tube lens. A short-pass dichroic mirror (FF670-SDi01, Semrock Inc, Rochester, NY) reflected two-photon excitation light onto the sample and allowed the detection of emitted fluorescence by the camera.

Single-photon stimulation of opsins was performed at 488 nm or 491 nm with a laser (MLD or Calypso respectively, COBOLT, Solna, SE) and a multimode fiber (core diameter 200 µm, 0.22 NA, QMMJ-3X-UVVIS-200/240-0.4-6, OZ Optics Ldt, Ottawa, CA). The laser was coupled to the fiber via a 10X objective (MPLN10X, Olympus, Milan, IT). On-off control of illumination was performed directly with a TTL input to the laser driver or *via* an acousto-optic modulator (R23080-3-LDT, Gooch & Housego PLC, Liminster, UK). Light intensity was 0.2 - 6 mW at the fiber tip. During all the experiments, the optical fiber was positioned ~ 500 µm above the craniotomy, at an angle of ~ 30°.

Phase modulation for holographic illumination

The SLM was controlled by custom software in Labview (National Instruments Corp, Austin, TX). Phase masks corresponding to desired illumination patterns were generated with a Gerchberg-Saxton (GS) Iterative Fourier Transform Algorithm (Di Leonardo et al., 2007). Before each experiment, a calibration routine with sub-micrometric precision was performed to match the FOV acquired in raster scanning with the holographic projection plane at the sample. This process relied on a customized ImageJ plugin (modified from StackReg) (Schindelin et al., 2012) and through a TCP/IP communication protocol between the proprietary PrairieView software and the custom software. Extended shapes of arbitrary geometry were drawn on reference images acquired in raster scanning before each photostimulation session. Shapes were then transformed into binary masks and used as input for the GS algorithm to obtain the desired illumination patterns at the sample. For two-photon holographic illumination experiments, individual neurons were illuminated with an elliptical shape covering the cell body. Wavelength for two-photon holographic illumination was 920 nm. The non-modulated component of light (zero order) was shifted into an out-of-focus plane (below the objective focal plane) by moving the position of L_2 while keeping the modulated component in focus by imposing appropriate phase modulation on the SLM. The same phase modulation applied to the SLM was also used in the laser-scanning configuration to acquire reference images. Under the experimental conditions used in this study, the power of the zero order component was in the range 3-10% of the total power illuminating the sample. We did not block the zero order component along the optical path because this solution prevented stimulation of neurons located in a small region in the center of the FOV. Illumination power used for *in vivo* experiments was in the range 10-92 mW *per* cell, corresponding to an intensity range of 0.11-0.97 mW/μm^2 *per* cell (calculated on an average illuminated area of 95 μm^2).

Characterization of the profile of the illuminating holographic volume

Images in Figure 1B and Figure S2 were acquired with a CCD camera (ORCA R2, Hamamatsu, Milan, Italy) mounted on the holographic stimulation setup (Figure S2A). Two filters (FES0650, Thorlabs, Newton, NJ and FF01-520/35, Semrock Inc, Rochester, NY, F in Figure S2A) were placed in front of the camera for fluorescence imaging and the dichroic mirror (FF670-SDi01, Semrock Inc., Rochester, NY; D in Figure S2A) was used to deflect excitation light to the sample. Fluorescence was collected by imaging a subresolved ultrathin (~150 nm) fluorescent layer (Antonini et al., 2014) via the objective (Olympus 40x) and the tube lens under two-photon holographic illumination (λ = 920 nm). z-stacks were acquired by shifting the vertical position of the objective in 1 µm steps. Images were processed with Fiji software (Schindelin et al., 2012). To compute the axial and lateral extension of projected shapes, a Gaussian filter was applied (σ, 2 µm). The lateral extension of the projected shape was determined at the focal plane as the width of the intensity profile after image thresholding with the ISODATA algorithm. The axial FWHM was calculated on the axial fluorescence profile (corresponding – at every z position - to the average fluorescence inside circular regions centered on the illumination shape) normalized and fitted with a Gaussian function.

***In vivo* electrophysiological recordings**

Neurons were targeted by imaging the fluorescent reporter with the two-photon microscope while monitoring the pipette electrical resistance by applying brief voltage pulses. When the pipette tip and the target cell were in close contact one to the other, a negative pressure was imposed to the pipette in order to achieve the juxtasomal configuration (resistance > 20 MΩ). For whole-cell shadow-patch recordings (Kitamura et al., 2008), pipettes were filled with intracellular solution containing 140 mM K-gluconate, 8 mM NaCl, 1 mM $MgCl_2$, 10 mM HEPES,10 mM Tris-phosphocreatine, 2 mM Na_2ATP, 0.5 mM NaGTP, pH 7.2 with KOH, mixed with Alexa Fluor 488 (20 µM). The fluorescent dye was injected into the extracellular space via pressure injection while imaging with the two-photon microscope. Target neurons were visualized and identified as dark ‘shadows’ in the two-photon image. For both shadow-patch (Figure S3) and two-photon targeted patch (Figure 4D-I) recordings, experiments were performed in whole-cell current-clamp mode. To assess expression of functional excitatory opsins in each recorded neuron, we recorded the cell response to a brief single-photon stimulus (stimulus duration: 50 ms; power at fiber tip: 0.2 - 6 mW) in the cell attached configuration. Only neurons that showed spiking responses with brief latency (2 - 3 ms) and low jitter were kept for successive two-photon stimulation. GtACR2 functionality *in vivo* was assessed at the beginning of the experiment using single-photon illumination (stimulus duration: 500 ms; power at fiber tip: 0.2 - 6 mW) and observing efficient silencing of the spontaneous firing or of the firing evoked by current injection (50 - 400 pA) in whole-cell configuration. Access resistance and resting potential were monitored during the experiment. Series resistance was not compensated and data were not corrected for the liquid junction potential. Cells with average resting potential more depolarized than -55 mV were excluded from analysis. Electrical signals were amplified by a Multiclamp 700B, low-pass filtered at 2.6 kHz, digitized at 50 kHz with a Digidata 1440 and acquired with pClamp 10 (Axon instruments, Union City, CA). Electrophysiological traces were analyzed using Clampfit 10.4 software (Molecular Device, Sunnyvale, CA) and IgorPro (WaveMetrics, Portland, OR).

Slice electrophysiology

Acute cortical coronal slices were prepared from the neocortex of P26 - P49 animals. After inducing deep anesthesia with urethane (16.5 %, 1.65 g/kg), brain was quickly dissected and placed in an ice-cold cutting solution containing: 130 mM K-gluconate, 15 mM KCl, 0.2 mM EGTA, 20 mM HEPES, and 25 mM glucose, with pH adjusted to 7.4 with NaOH and oxygenated with O_2 100%. Slices (slice thickness: 300 μm) were cut with a vibratome (VT1000S, Leica Microsystems, GmbH, Wetzlar, Germany) and immersed for 1 min in solution at room temperature (RT) containing: 225 mM D-mannitol, 25 mM glucose, 2.5 KCl, 1.25 NaH_2PO_4, 26 $NaHCO_3$, 0.8 mM $CaCl_2$, 8 mM $MgCl_2$, pH 7.4 with 95% O_2/5% CO_2. Slices were then incubated for 30 min at 35°C in standard ACSF (sACSF) composed of: 125 mM NaCl, 2.5 mM KCl, 25 mM $NaHCO_3$, 1.25 mM NaH_2PO_4, 2 mM $MgCl_2$, 1 mM $CaCl_2$, 25 mM glucose, pH 7.4 with 95% O_2/ 5% CO_2. After incubations slices were maintained in sACSF at RT until use. During photostimulation experiments, slices were positioned in submerged recording chamber (RC#, Warner Instruments, Hamden, CT, USA) and continuously perfused with fresh bathing solution (125 mM NaCl, 2.5 mM KCl, 25 mM $NaHCO_3$, 1.25 mM NaH_2PO_4, 2 mM $MgCl_2$, 2 mM $CaCl_2$, 25 mM glucose, pH 7.4 with 95 %O_2/5 % CO_2) maintained at 30 - 32 °C by an inline solution heater (TC-344B, Warner Instruments, Hamden, CT, USA). The same objective (Olympus 40x, 0.8 NA) was used for *in vivo* electrophysiology and slice experiments. Pipettes (pipette resistance: 3 - 4 MΩ) were filled with intracellular solution containing: 140 mM K-gluconate, 8 mM NaCl, 1 mM $MgCl_2$, 2 mM Na_2ATP, 0.5 mM NaGTP, 10 mM HEPES, 10 mM Tris-phosphocreatine to pH 7.2 with KOH. Alexa Fluor 488 (20 μM) was added to the intracellular solution to allow identification of patched neurons under two-photon illumination. Pipette guidance during patch-clamp recordings was performed using infrared differential interference contrast. After the establishment of the whole-cell configuration, functional expression of GtACR2 was first verified using brief single-photon light pulses (pulse duration: 500 ms; power at the fiber tip: 0.2 - 6 mW). For holographic two-photon illumination, a high resolution image of the recorded neuron filled with Alexa Fluor 488 was acquired with the two-photon microscope in raster scanning and an extended elliptical shape was projected to the cell body of the recorded neuron (stimulus duration: 500 ms). Currents evoked by holographic illumination were recorded in voltage-clamp at -50 mV. Access resistance, resting potential and injected current necessary to maintain the recorded neuron at -50 mV were monitored during the experiment. Series resistance was not compensated and data were not corrected for the liquid junction potential. Cells with average resting potential more depolarized than -55 mV were excluded from analysis. Voltage-clamp recorded currents were low-pass filtered at 2 kHz, digitized at 10 kHz with the same instrumentation used for *in vivo* recordings.

Analysis of electrophysiological recordings

For the analysis of *in vivo* whole-cell recordings from GtACR2$^+$ cells, action potential (AP) firing frequency was calculated during injection of positive current steps (current amplitude: 50-100 pA) in a time window before (Pre; window duration: 0.4 s), during (Stim; window duration: 0.5 s) and after (Post; window duration: 0.4 s) holographic stimulation over 15 - 120 stimulation trials. Δ_{AP}Freq and the spatial resolution were calculated as for juxtasomal electrophysiological recordings (considering -$_{AP}\Delta$Freq). Illumination power levels used for resolution measurements in GtACR2$^+$ neurons were set to obtain less than 100% inhibition (≤ 80 mW). A cell was considered responsive to two-photon holographic illumination if Δ_{AP}Freq was < -0.1 of the average firing frequency in Pre and Post periods at power values ≤ 80 mW *per* shape and current injection of 50-100 pA. 14 out of 15 GtACR2-positive cells were considered as two-photon responsive. In slice experiments, the amplitude of GtACR2 photocurrents was calculated as the peak current in the first 100 ms after light onset on a current trace obtained by averaging 3 - 7 stimulation trials. Data from electrophysiological recordings in cultured neurons were analyzed using custom scripts written in Matlab (Mathworks). To quantify currents in response to two-photon scanning, holding current traces were filtered with a second order Butterworth infinite impulse response bandpass filter (half power frequencies: 49 Hz, 51 Hz) and a Savitzky-Golay 11-point, second order, Welch window function filter. The reported N refers to the number of recorded neurons. Escaped APs during full-field illumination were removed for presentation purposes.

Analysis of jRCaMP1a signals during holographic stimulation at 920 nm

Temporal series for Figure S7A-H were acquired in the raster scanning configuration during spontaneous activity in anesthetized mice (40x objective, 100 x 100 pixels, frame rate, 11 Hz; pixel dwell time, 4 μs; λ = 1100 nm; imaging power, 15-32 mW) while simultaneously delivering repeated two-photon holographic stimuli (λ = 920 nm) to one imaged neuron located in the center of the FOV by projecting an elliptical shape covering the cell body of the target neuron. Two different protocols were used: 8 stimuli of 500 ms duration at 0.1 Hz and 5 stimuli of 5 s duration at 0.05 Hz. The average stimulation power of the holographic illumination was 30 mW or 50 mW. Target cortical neurons were located 130 - 290 μm deep in the cortex. Temporal series were imported into ImageJ/Fiji software for analysis. Ring shaped ROIs defining neuronal somata were manually selected. For each recording, the time course of the average fluorescence from the ROI corresponding to illuminated neuron and the average background fluorescence were computed (background was defined as the portion of the image which did not contain ROIs). Background subtraction was adopted to correct for neuropil contamination (Chen et al., 2013) and for artifacts induced by holographic illumination. For the analysis in Figure S7E, ten

frames of baseline jRCaMP1a fluorescence not comprising spontaneous transients were considered in a 5 s time window before (Pre), during (Stim) or after (Post) holographic illumination for each repetition of the illumination pulse and then averaged. For the analysis in Figure S7G, ten frames of baseline jRCaMP1a fluorescence were considered before the first illumination stimulus (Start) and after the 8th illumination stimulus (End). Baseline jRCaMP1a fluorescence for the Mid time point was defined as the average baseline between the 3rd and the 6th light stimuli. For the analysis in Figure S7H, ten frames of baseline jRCaMP1a fluorescence were considered before the first illumination stimulus (Start) and after the 5th illumination stimulus (End). Baseline jRCaMP1a fluorescence for the Mid time point was defined as the average baseline between the 2nd and the 4th stimuli. For experiments in Figure S7I-M, temporal series from jRCaMP1a expressing neurons located in layer 2/3 (cell depth, 150 – 220 µm) of the barrel cortex were acquired as for data in Figure S7A-H. Contralateral whiskers were periodically (0.07 Hz) stimulated directing brief (stimulus duration: 300 ms) air puffs toward the vibrissae through a borosilicate pipette connected to a pneumatic pressure injector (Intracel, Royston Herts, UK). Only on even trials, whisker stimuli were coupled with simultaneous two-photon holographic illumination (λ = 920 nm, 5s duration) of the whisker-responsive neuron. The change in fluorescence for background subtracted traces relative to the baseline ($\Delta F/F_0$) was computed as a function of time, with the fluorescence baseline (F_0) calculated in ten frames before each whisker stimulation. Only couples of reliable responses (Peak $\Delta F/F_0 > 0.2$) were considered for further analysis. The decay time constant (τ_{off}) was calculated by fitting the descending phase of whisker evoked responses with an exponential decaying function.

Experiments in Figure 7 were performed on PV-cre mice co-injected with AAVs carrying flex.jRCaMP1a (AAV1syn.flex.NES.jRCaMP1a or AAV1CAG.flex.NES.jRCaMP1a) and AAV2/1. hSyn1.SIO.GtACR2-ts-fRed-Kv2.1.WPR (Mahn et al., 2017). Due to the dim fRed fluorescence, in a subset of mice the AAV carrying SIO.GtACR2-ts-Fred-Kv2.1 was co-injected with AAV2/1.EF1a.DIO.eYFP.WPRE, in order to better visualize opsin-expressing cells. Four to seven weeks after injection mice were used for experiments. Two-photon imaging in anesthetized mice was performed in layer 2/3. A reference image of the selected FOV was acquired and a shape covering the soma of a target interneuron was generated by the SLM (stimulation wavelength, λ = 920 nm) and projected at the sample. Temporal series were simultaneously acquired in raster scanning configuration with the imaging beam (40x objective; 100 x 100 pixels; frame rate: 11 Hz; pixel dwell time: 4 µs; λ_{exc} = 1100 nm, power range: 20 - 34 mW). Holographic photostimulation duration was 5 s and was repeated at 0.05 Hz for 5 times (stimulation power: 50 mW). In a subset of experiments during two-photon imaging and holographic inhibition, the spiking activity of imaged PV^{+} interneurons was simultaneously recorded with a patch pipette in the juxtasomal configuration. jRCaMP1a signals were analyzed with ImageJ/Fiji software as described above. AP firing frequency was calculated in a time window before (Pre; window duration: 5 s), during (Stim; window duration: 5 s) and after (Post; window duration: 5 s) holographic stimulation. Baseline jRCaMP1a fluorescence was calculated in a time window before (Pre; 1 s long, starting 4 s before stimulation), during (Stim; 1 s long, starting 1s before the end of the light stimulus) and after (Post; 1 s long, starting 9 s after the end of the stimulus) holographic stimulation. The area underneath jRCaMP1a fluorescence traces was calculated in a time window before (Pre; 4 s long, before stimulation), during (Stim; 5 s long) and after (Post; 4s long, starting 6 s after the end of the light stimulus) holographic stimulation and normalized to the corresponding time interval.

Simultaneous jRCaMP1a imaging and juxtasomal electrophysiological recording

Temporal series from jRCaMP1a expressing neurons were acquired in anesthetized mice in the raster scanning configuration (40x objective, 100 x 100 pixels, frame rate: 11 Hz or 48 x 48 pixels, frame rate: 33 Hz; pixel dwell time, 4 µs; λ_{exc} = 1100 nm) while simultaneously recording the spiking activity of the imaged neuron with a patch pipette for 1 – 3 minutes. For each series, ring-shaped ROIs defining neuronal somata were manually selected and neuropil contamination was subtracted. To quantify the accuracy for detecting single APs or bursts, we identified putative burst initiators (BI) in the electrophysiological trace as APs isolated by previous APs by more than 1s. Every BI triggered an integration window of 350 ms in which the number and timing of spikes were recorded. Integration window length was chosen to reflect the accumulation of calcium signal for jRCaMP1a (Dana et al., 2016). According to the total number of spikes in the window, 1-5 AP events were classified. Rare events with more than 5 spikes were discarded. For each event in a given n-AP class (1-5AP), fluorescence traces consisting of 20 frames at 33 Hz (or 7 frames at 11 Hz) before (F_{0i}) and 34 frames (or 11 frames at 11 Hz) after the *i*th BI were assembled in 54-dimensional vectors (or 18-dimensional for 11 Hz), $(\Delta F/F_0)_i$. Segments of noisy traces with the same number of frames were taken (one for each trace) from periods without APs, $(\Delta F^{noise}/F_0)_j$. For each n-AP class, the average of all $(\Delta F/F_0)_i$ traces was normalized and used as a template vector $\Delta F/F_0$. The scalar product of $(\Delta F/F_0)_i$ or $(\Delta F^{noise}/F_0)_j$ with the template $\Delta F/F_0$ was calculated to obtain a scalar f_i or n_j, respectively (Chen et al., 2013). The AP detection threshold for each n-AP class was defined as the 95th percentile of all n_j values and the percentage of the f_i values above the detection threshold was the n-AP detection accuracy (Dana et al., 2016). For each 2-5AP event, average firing frequency was calculated as the inverse of the average inter-spike-interval. Peak $\Delta F/F_0$ was calculated on both

detected and undetected events at the time point corresponding to the maximum component of the template vector $\Delta F/F_0$. Imaging power: 20 - 40 mW at 1100 nm excitation wavelength. Neurons recorded in layer 2/3 at depth 110 - 190 µm.

Confocal image acquisition and analysis

Deeply anesthetized animals were transcardially perfused with 0.01 M PBS (pH 7.4) followed by 4 % paraformaldehyde. Brains were post-fixed for six hours in the same solution and cryoprotected with a 30 % sucrose solution in 0.1 M phosphate buffer (pH 7.4). Brains were collected in embedding molds, covered with Tissue-Tek O.C.T. Compound (Sakura Finetek EuropeB.V.) and sectioned with a cryostat (Leica Microsystems, Milan, IT). Coronal sections (slice thickness, 40 µm) were serially collected in multiwell dishes and counterstained with Hoechst (1:400, Sigma Aldrich, Milan, IT). Sections were mounted, dried, and coverslipped with a DABCO [1,4-diazobicyclo-(2,2,2)octane]-based antifade mounting medium. High-resolution confocal z-stacks (1,5 µm steps; 2048 x 2048 pixels, 40x objective) were acquired with a Leica SP5 inverted confocal microscope (Leica Microsystems, Milan, IT) in order to estimate density (neurons/mm^3) of opsin-expressing (opsin$^+$) neurons for the different conditions tested in this study: opsin$^+$ neurons in cortical layer 2/3 of mice injected with AAV1.hSyn.hChR2(H134R).EYFP, AAV1.hSyn.hChR2(H134R).eYFP.Kv2.1 or AAV2/1.hSyn.GtACR2.eGFP; opsin$^+$ interneurons in layer 2/3 of SST-cre or PV-cre mice injected with AAV1-EF1a-dFlox-hChR2(H134R)-mCherry-WPRE-hGH, and opsin$^+$ neurons located in upper layer 4 (300 – 450 µm depth) of Scnn-cre x tdTomato mice injected with AAV1-EF1a-DIOhChR2(H134R)-eYFP-WPRE-hGH (both visual and barrel cortices were considered). Two coronal slices *per* animal with visible opsin expression in layer 2/3 and 4 were randomly chosen in a rough volume (~1.5 mm radius) around the injection site. 2-3 z-stacks *per* coronal slice were acquired (N = 10 z-stacks from 2 mice for ChR2-eYFP; N = 12 z-stacks from 3 mice for the soma-targeted ChR2-eYFP, layer 4 ChR2-eYFP, SST-cre, and PV-cre; N = 12 z-stacks from 2 mice for GtACR2-eGFP). For a given z-stack, random sampling was performed by applying a virtual counting grid (square's size 85 µm x 85 µm for layer 2/3 neurons, 104 µm x 104 µm for interneurons and 66 µm x 66 µm for layer 4 neurons) over the whole area of interest using ImageJ (Fiji.sc) and counting opsin$^+$ cells throughout the whole thickness of the tissue slice (40 µm) in five randomly chosen squares of the grid. Opsin$^+$ neurons were identified based on the signal of the fluorescence tag fused to the opsin. Cellular identity of opsin$^+$ neurons was confirmed by looking at the Hoechst staining. The half activation volume ($V_{1/2}$) in Table S2 was calculated as the sum of the volumes of two half-ellipsoids, with semi-axes ($l_{1/2}$ rad., $l_{1/2}$ rad., and $l_{1/2}$ ax. up) and ($l_{1/2}$ rad., $l_{1/2}$ rad., and $l_{1/2}$ ax. down). The number of additional opsin positive neurons, besides the target cell, contained in $V_{1/2}$ was calculated from the average cell density and the average value of the volume of half activation. Confocal high-resolution images (2048 x 2048 pixels; Leica SP5, Wetzlar, DE) were used for the analysis of opsin expression in neurites. Coronal slices with visible opsin expression were randomly chosen in a rough volume (~1.5 mm radius) around the injection site. Confocal images of sparsely labelled neurons were acquired near the edge of the cortical area expressing the transgene (or in the non-injected hemisphere). z-stacks with 1 µm steps containing 1-3 neurons were acquired. The image analysis was performed in ImageJ on z-projection of ~10 sequential planes. Only apical dendrites roughly coplanar with the coronal section were considered for the analysis. For each neuron, fluorescence analysis was performed as in (Shemesh et al., 2017) to determine ChR2-eYFP signal intensity as a function of the distance from the soma. Intensity values were normalized to the ChR2-eYFP intensity at the soma.

Supplemental Discussion

High spatial resolution two-photon optogenetic excitation of multiple neurons simultaneously can be obtained with various methods. In one approach commonly named "spiral scan" configuration, a SLM is used to multiplex the laser beam and divide it into several different beamlets each addressing one of the neurons of interest. All beamlets are then scanned using a spiral trajectory across the cell bodies of the target neurons (Packer et al., 2013; Packer et al., 2015). Alternatively, an extended circular shape approximately the size of a cell body can be obtained by underfilling the back-aperture of the objective, effectively reducing its numerical aperture. This shape can then be sequentially projected to the target neurons (Rickgauer et al., 2014). The perturbation methods involving an extended shape that is projected to the cell body of the target neuron is appealing because it allows simultaneous activation of the light-sensitive opsins that are somatically expressed and may lead to larger instantaneous photocurrents and more efficient perturbation of neuronal excitability compared to sequential approaches. Digital holography has been efficiently used to dynamically generate extended shapes of illumination (Chaigneau et al., 2016; Papagiakoumou et al., 2010; Papagiakoumou et al., 2013). One advantage of this approach is that it allows the generation of multiple extended regions of interest within a FOV, enabling simultaneous (and not sequential) stimulation of ensembles of neurons. Previous studies demonstrated that this approach can be efficiently applied to increase cellular excitability in the rodent brain *in vitro* (Papagiakoumou et al., 2010; Papagiakoumou et al., 2013; Ronzitti et al., 2017; Shemesh et al., 2017) and *in vivo* (Pegard et al., 2017). In this study, we demonstrate the applicability of holographic illumination for high spatial resolution bidirectional perturbation of neurons in the mouse neocortex *in vivo*. We found that holographic stimulation of ChR2-expressing cells efficiently drives neurons to spike (Figures 1 and 2), while holographic illumination of GtACR2-expressing neurons reliably decreases their firing rate (Figure

4D-H). Importantly, we demonstrate that this approach is generalizable and can be applied to cell types that differ in biophysical properties, morphological structure and anatomical location (Figure 3). This is necessary to allow for the application of our method to investigate the role of precise spatiotemporal patterns of neural activity in driving higher cortical functions, as activity patterns are distributed in space and time across several cellular subtypes (Carrillo-Reid et al., 2017). In comparing the results of holographic stimulation in different cell types, it is important to consider that the spatial resolution of photostimulation is not only influenced by the morphology of the cell and by the size of the excitation volume but also by the density of opsins in the plasma membrane, Moreover, while previous work (Carrillo-Reid et al., 2017; Karnani et al., 2016; Packer et al., 2015; Rickgauer et al., 2014) demonstrated two-photon manipulation of neurons in superficial (i.e., layer 2/3) cortical layers *in vivo*, we showed that this approach can also access deeper layers (i.e., layer 4) in the intact brain. This will allow manipulating activity patterns with high spatial resolution in the main thalamorecipient cortical lamina and study how these patterns are processed as they flow through the complex circuitry of the cortical column.

The spatial resolution and efficiency of stimulation may be further improved by implementing temporal focusing and by reducing inhomogeneities within the extended shape (i.e., speckles). For example, temporal focusing may be used to improve the axial resolution of excitation (Begue et al., 2013; Oron et al., 2005; Papagiakoumou et al., 2013). Moreover, more efficient methods to restrict opsin expression to the cell soma may further increase the spatial precision of holographic illumination. Finally, to improve the excitation efficiency while maintaining reduced cross-talk between imaging and photostimulation, blue light-sensitive opsins with larger conductance compared to ChR2, as for example Chronos (Ronzitti et al., 2017) or CoChR (Shemesh et al., 2017), could be used in combination with jRCaMP1a.

The key steps of synaptic signal integration that lead to shaping the AP output of a neuron largely occur at the dendritic level (Stuart et al., 2015). In contrast, patterned two-photon illumination methods as the one presented in this study modulate the AP firing rate by generating photocurrents mostly in the somatic compartment. This fundamental difference needs to be taken into consideration when considering all-optical approaches as the method of choice. However, it must also be considered that if somatic photocurrents are kept below the threshold for AP generation, they only increase or decrease the neuron's probability of firing which is ultimately dictated by the natural process of synaptic integration across all cellular compartments. It is also worth underlying that for all-optical imaging and manipulation methods to be efficiently applied for the dissection of the cellular mechanisms underlying brain function, data handling and the development on new and more advanced algorithms (Panzeri et al., 2017) for the analysis of circuit activity are of crucial importance.

We found that targeting ChR2 to the soma (Figure 2) significantly increased the average spiking response in the illuminated neuron *in vivo*. This result is in line with previous electrophysiological evidence *in vitro* (Baker et al., 2016) suggesting that an increase in the expression of channels in the somatic and perisomatic compartment occur when a soma-targeted opsins is expressed. Moreover, we found that the axial spatial resolution to be significantly increased in neurons expressing soma-targeted ChR2 compared to neurons expressing non soma-targeted ChR2 (Table S1-S2). In principle, a larger number of ChR2 channels in the membrane would decrease the spatial resolution because it increases the probability of stimulating with the tail of the illumination volume. In contrast, the restriction of opsins localization to the somatic compartment would have the opposite effect. Thus, the observation that the axial spatial resolution is higher in neurons expressing the soma-targeted ChR2 suggests that the second mechanism is preponderant under our experimental conditions, in line with previous observation using the same construct *in vitro* (Baker et al., 2016).

The choice of a red-shifted calcium indicator and a blue light-sensitive opsin significantly decreased the cross talk between imaging and opsin activation (Figure 5, Figure S1). This requires a significant separation of the two-photon excitation spectra of the opsin from that of the indicator. However, simultaneous imaging and photostimulation of opsins with absorption spectrum largely overlapping with that of the functional indicator has been performed, e.g. (Baker et al., 2016; Dal Maschio et al., 2017). Thus, optimizing protein expression level and light power values used for imaging may decrease opsin activation during imaging under conditions in which the opsin shows significance absorbance at the imaging wavelength.

Utilizing low repetition rate lasers for holographic stimulation (Chaigneau et al., 2016; Pegard et al., 2017; Ronzitti et al., 2017; Shemesh et al., 2017) decreases the average stimulation power *per* cell, the latency of neural spiking response, and the jitter of evoked spikes beyond what has been achieved in the current study. The use of low repetition rate lasers might thus be important for optogenetic stimulation when the experimental question necessitates the precise temporal control over patterns of spiking activity in brain circuits. This might be the case for instance for a "virtual sensation" experiment, in which the aim is to reproduce a sensory perception in the absence of the sensory input by driving activity patterns with high temporal (and spatial) precision (Panzeri et al., 2017). It is important to note that sub-millisecond precision in stimulating

AP firing using low repetition rate lasers and holographic approaches similar to the one described in this study was obtained both using fast opsins (e.g., Chronos) (Ronzitti et al., 2017) and slower opsins (e.g., CoChR) (Shemesh et al., 2017). In this regard, the membrane time constant and the instantaneous photocurrent amplitude (thus a large instantaneous power of the light pulse) are the main limiting steps for the time domain of photostimulation, more than the opsin kinetics. To obtain high temporal precision in stimulating cells with complex AP patterns high refresh rate SLMs are also needed. Current technologies allow changing the stimulation pattern with delays that range from few ms to tens of ms (Thalhammer et al., 2013). When network dynamics are investigated at this high temporal resolution one of the main limitation is also represented by the slow kinetics which characterize most genetically encoded calcium indicators (Chen et al., 2013; Dana et al., 2016). Further improvement of these calcium indicators or the combination of efficient voltage-sensitive dyes imaging (Knopfel et al., 2015) and patterned illumination might obviate to this limitation. However, there are other scenarios where this temporal precision might not be needed. For example, when natural network dynamics induced by a sensory stimulus need only to be "biased" by slightly increasing or decreasing the probability of firing of specific ensembles of neurons (Panzeri et al., 2017). Our system represents an efficient technical solution to be applied to this stochastic perturbative approach.

Supplemental References

Antonini, A., Liberale, C., and Fellin, T. (2014). Fluorescent layers for characterization of sectioning microscopy with coverslip-uncorrected and water immersion objectives. Opt.Express *22*, 14293–14304.

Begue, A., Papagiakoumou, E., Leshem, B., Conti, R., Enke, L., Oron, D., and Emiliani, V. (2013). Two-photon excitation in scattering media by spatiotemporally shaped beams and their application in optogenetic stimulation. Biomed.Opt.Express *4*, 2869–2879.

Beltramo, R. et al. (2013). Layer-specific excitatory circuits differentially control recurrent network dynamics in the neocortex. Nat.Neurosci. *16*, 227–234.

Di Leonardo, R., Ianni, F., and Ruocco, G. (2007). Computer generation of optimal holograms for optical trap arrays. Opt.Express *15*, 1913–1922.

Karnani, M.M., Jackson, J., Ayzenshtat, I., Tucciarone, J., Manoocheri, K., Snider, W.G., and Yuste, R. (2016). Cooperative Subnetworks of Molecularly Similar Interneurons in Mouse Neocortex. Neuron *90*, 86–100.

Kitamura, K., Judkewitz, B., Kano, M., Denk, W., and Hausser, M. (2008). Targeted patch-clamp recordings and single-cell electroporation of unlabeled neurons in vivo. Nat Methods *5*, 61–67.

Knopfel, T., Gallero-Salas, Y., and Song, C. (2015). Genetically encoded voltage indicators for large scale cortical imaging come of age. Curr.Opin.Chem.Biol. *27*, 75–83.

Mahn, M., Prigge, M., Ron, S., Levy, R., and Yizhar, O. (2016). Biophysical constraints of optogenetic inhibition at presynaptic terminals. Nat Neurosci. *19*, 554–556.

Oron, D., Tal, E., and Silberberg, Y. (2005). Scanningless depth-resolved microscopy. Opt Express *13*, 1468-1476.

Packer, A.M., Roska, B., and Hausser, M. (2013). Targeting neurons and photons for optogenetics. Nat Neurosci. *16*, 805–815.

Pegard, N.C., Mardinly, A.R., Oldenburg, I.A., Sridharan, S., Waller, L., and Adesnik, H. (2017). Three-dimensional scanless holographic optogenetics with temporal focusing (3D-SHOT). Nat Commun. *8*, 1228.

Schindelin, J. et al. (2012). Fiji: an open-source platform for biological-image analysis. Nat Methods *9*, 676–682.

Shemesh, O.A., Tanese, D., Zampini, V., Linghu, C., Piatkevich, K., Ronzitti, E., Papagiakoumou, E., Boyden, E.S., and Emiliani, V. (2017). Temporally precise single-cell-resolution optogenetics. Nat Neurosci. *20*, 1796–1806.

Stuart, G.J. and Spruston, N. (2015). Dendritic integration: 60 years of progress. Nat Neurosci. *18*, 1713–1721.

Thalhammer, G., Bowman, R.W., Love, G.D., Padgett, M.J., and Ritsch-Marte, M. (2013). Speeding up liquid crystal SLMs using overdrive with phase change reduction. Opt.Express *21*, 1779–1797.
